# Construction and comparison of gene co-expression networks shows complex plant immune responses

**DOI:** 10.7717/peerj.610

**Published:** 2014-10-09

**Authors:** Luis Guillermo Leal, Camilo López, Liliana López-Kleine

**Affiliations:** 1 Department of Statistics, Universidad Nacional de Colombia, Bogotá, Colombia; 2 Department of Biology, Universidad Nacional de Colombia, Bogotá, Colombia

**Keywords:** Gene co-expression networks, Similarity measures, Similarity threshold, Principal Component Analysis, Networks comparison, Plant immunity

## Abstract

Gene co-expression networks (GCNs) are graphic representations that depict the coordinated transcription of genes in response to certain stimuli. GCNs provide functional annotations of genes whose function is unknown and are further used in studies of translational functional genomics among species. In this work, a methodology for the reconstruction and comparison of GCNs is presented. This approach was applied using gene expression data that were obtained from immunity experiments in *Arabidopsis thaliana*, rice, soybean, tomato and cassava. After the evaluation of diverse similarity metrics for the GCN reconstruction, we recommended the mutual information coefficient measurement and a clustering coefficient-based method for similarity threshold selection. To compare GCNs, we proposed a multivariate approach based on the Principal Component Analysis (PCA). Branches of plant immunity that were exemplified by each experiment were analyzed in conjunction with the PCA results, suggesting both the robustness and the dynamic nature of the cellular responses. The dynamic of molecular plant responses produced networks with different characteristics that are differentiable using our methodology. The comparison of GCNs from plant pathosystems, showed that in response to similar pathogens plants could activate conserved signaling pathways. The results confirmed that the closeness of GCNs projected on the principal component space is an indicative of similarity among GCNs. This also can be used to understand global patterns of events triggered during plant immune responses.

## Introduction

Molecular biological high-throughput techniques have provided a great amount of diverse and informative gene expression data, currently available in genomic databases. These data, if properly analyzed, allow for a better understanding of the biological processes in different organisms. The construction of functional gene networks that are based on gene expression data are termed gene co-expression networks (GCNs), which reflect information based on the relationships between genes (and/or the proteins they encode) that indicate a coordinated participation in a common biological process or pathway (Atias, Chor & Chamovitz, 2009; Hwang et al., 2011). GCNs predict functional annotations for genes whose function is unknown (Ficklin & Feltus, 2011). Some studies have also confirmed through experimental validation that the predictions are accurate (Seo et al., 2011).

Several methodologies have been used for the construction of GCNs in plants in order to understand important biological processes (López-Kleine, Leal & López, 2013), trying to represent as much information as possible using gene expression data from heterogeneous experiments (Atias, Chor & Chamovitz, 2009). Most of these methodologies share four main steps that are solved in different manners: (1) gene expression data selection and the construction of expression matrices, (2) the selection of a similarity measurement and the construction of gene similarity matrices (Butte & Kohane, 2000; Mahanta et al., 2012), (3) similarity threshold selection (Elo et al., 2007; Luo et al., 2007) and (4) the comparison of GCNs that were obtained from different samples or species, as has been proposed as the final step by several works (Elo et al., 2007; Skinner et al., 2011).

The confidence in the obtained GCNs depends on the reliability and objectiveness of the approach used at each of these steps. Additionally, when heterogeneous gene expression samples are used in conjunction, special care is required to maintain a high signal/noise ratio. Selecting a similarity metric that captures the relationship between gene expression profiles is the first critical decision in the methodology (Zhang & Horvath, 2005). The Pearson Correlation Coefficient (PCC) is the most used similarity metric due to its simple implementation and appropriateness for this task (Edwards et al., 2010; Ouyang et al., 2012). Nevertheless, as expression profiles can be correlated non-linearly, many genes with an interesting coordinated co-expression are not retained for inclusion in the final GCN using PCC (Bandyopadhyay & Bhattacharyya, 2011). Furthermore, the PCC is affected by outlying observations that originate pairs of genes that are co-expressed incorrectly (Mutwil, 2010). Studies have confirmed that the PCC is high even if genes are neither overexpressed nor underexpressed across conditions (Bandyopadhyay & Bhattacharyya, 2011) and that it also fails in the detection of proximity between expression profiles (Mahanta et al., 2012). Several metrics have been introduced to detect any dependence between expression profiles while enhancing the robustness if noisy data are available (Numata, Ebenhöh & Knapp, 2008; Bandyopadhyay & Bhattacharyya, 2011). Metrics that are based on information theory, such as the Non-linear Correlation coefficient based on Mutual Information (NCMI), perform well with expression data, due to the lack of distribution assumptions and the fact that these metrics are not affected by data transformations (Numata, Ebenhöh & Knapp, 2008). Recently, the Normalized Mean Residue Similarity (NMRS) showed good performance in detecting shifted patterns of expression profiles (Mahanta et al., 2012). An evaluation of these metrics compared to the PCC is essential to establish their strengths or weaknesses in capturing functional lineal and non-lineal relationships between genes.

Once an appropriate similarity measure has been applied, the second step is selecting the similarity threshold. Selecting a similarity threshold is a decision that frequently relies on subjective criteria or previous biological knowledge (Ala et al., 2008). Elaborated approaches for selecting the threshold objectively have been proposed (Nayak et al., 2009). Methods based on the clustering coefficient of graphs (Elo et al., 2007), spectral graph theory (Perkins & Langston, 2009) and random matrix theory (Luo et al., 2007) attempt to differentiate true co-expressed genes from random noise. In these methods, the structure of GCNs is revealed in a systematic way without subjective intervention (Luo et al., 2007). However, their complexity and dependence on assumptions makes them restrictive. Among these methods, clustering coefficient-based methods are robust and intuitive (Elo et al., 2007).

Regarding the comparison of networks as a final step in most of the studies constructing GCNs, some strategies aim to study conserved topological or biological information between GCNs (Mutwil et al., 2011). The comparison of networks using graph variables and multivariate approaches has also been developed (Costa et al., 2005; Elo et al., 2007). Only topological or spectral variables are used to characterize networks, therefore, genomic information is not reflected in graph properties, and biological conclusions are not revealed. An efficient strategy to characterize and compare GCNs based on a multivariate analysis, allowing researchers to include and also obtain valuable genomic data from networks and to infer global similarities, is still not available.

In the present work, we constructed GCNs based on gene expression data that were obtained from plant immunity experiments. The plants represent an important source of nutrients for most organisms. To gain access to these nutrients, pathogens have to survive the plant responses. Plant immunity has been classified into two branches according the molecules involved in the recognition (Jones & Dangl, 2006). The first branch depends on the recognition of microorganism-associated molecular patterns (MAMPs) by pattern recognition receptors (PRRs). This immunity is named MAMP-triggered immunity (MTI also known as PTI) (Zipfel, 2009). The second branch of plant immunity depends on the recognition of pathogen effector proteins, which are translocated and recognized in the plant cytoplasm by resistance (R) proteins. This branch has been called effector-triggered immunity (ETI) (Jones & Dangl, 2006). The PTI and/or ETI induce a systemic acquired resistance (SAR) that confers a broad-spectrum and long-term resistance (Durrant & Dong, 2004). The recognition of MAMPs or effectors triggers a diverse array of responses, including ion fluxes, the production of reactive oxygen species (ROS) and the activation of MAP kinase signaling pathways, leading to the activation of transcription factors that in its turn modulate the host gene expression (Dodds & Rathjen, 2010). The changes (induction and repression) in gene expression during different plant immune responses have been studied in several plant pathosystems (Glazebrook, 2005; Birkenbihl & Somssich, 2011), but *Arabidopsis thaliana*-*Pseudomonas syringae* remain the primary models for the study of plant–pathogen interactions (Nishimura & Dangl, 2010).

In the present work, we performed the four steps of GCN construction, carefully evaluating the statistical robustness and objectivity during each step. The careful selection of the best method and some improvements during the threshold selection step allowed us to obtain a general picture of gene expression reprogramming during plant pathogen immunity through the GCN construction. Pathogen resistance microarray datasets from *Arabidopsis*, rice (*Oryza sativa*), soybean (*Glycine max*), tomato (*Solanum lycopersicum*) and cassava (*Manihot esculenta*) were used. We evaluated the performance of the Absolute value of the Pearson Correlation Coefficient (APCC) against two metrics, NCMI and NMRS. For the similarity threshold selection, a modification of the clustering coefficient-based method is proposed to select the similarity thresholds. For the comparison step, the GCNs were characterized and a Principal Component Analysis was performed. The GCNs were clustered based on the principal component (PC) space using the K-means clustering algorithm. We found that the distance between the GCNs in the PC space can be used to analyze their structural and functional similarities within and between species. The comparative analyses allowed for the identification of common elements, indicating cross-talk between the different signaling responses to pathogens in the studied plant species.

## Materials & Methods

### Expression matrices construction

Pathogen resistance microarray data was used in this work. GEO DataSet repositories were queried for the expression data from microarray experiments (http://www.ncbi.nlm.nih.gov/geo/). A total of 40 non-processed datasets for *Arabidopsis thaliana*, 8 for rice, 5 for soybean and 3 for tomato were collected. Three cassava microarray datasets were obtained from previous studies (López et al., 2005).

The datasets were independently pre-processed through noise reduction, normalization and log2 transformation. The Robust Multiarray Average (RMA) method (Bolstad et al., 2003) was applied to Affymetrix data using the R affy library (R Development Core Team, 2011), while the two-color microarray data were pre-processed using the marray and Agi4x44PreProcess libraries.

The probe IDs were converted into gene IDs using a conversion table for each platform. Single probes that matched more than one gene were removed. For those multiple probes that matched a single gene, the maximum expression was assigned to the gene.

A filter of the samples and genes was applied to the datasets to reduce missing data. First, a common gene list was obtained, and those samples representing less than 50% of the common genes were removed. Afterwards, those genes that were represented in less than 75% of the total samples were removed.

**Figure 1:**
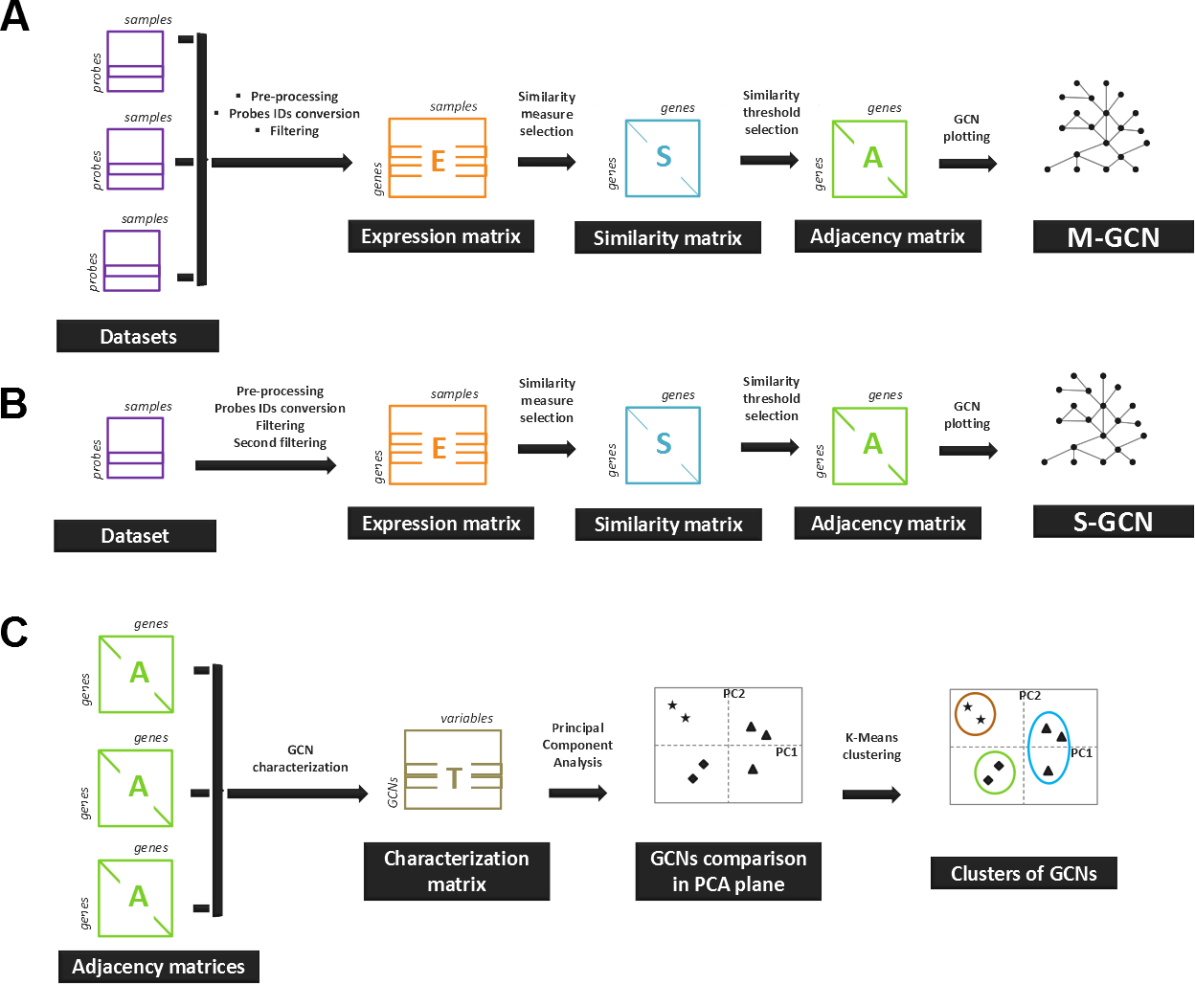
The overall steps for the construction and comparison of the GCNs. (A) The expression data from several microarray experiments were pre-processed and merged into a single expression matrix. Then, a similarity measurement was used to calculate a similarity matrix. A similarity threshold was chosen, and the adjacency matrix was calculated. The resulting GCN was termed a multiple-experiment GCN (M-GCN). (B) The expression data from a single microarray experiment were processed to assemble the expression matrix. The remaining steps were executed as in (A). The resulting GCN was termed a single-experiment GCN (S-GCN). (C) The adjacency matrices from the GCNs were characterized with the graph variables. The characterization based on network variables was constructed, and the PCA was used to compare the GCNs.

At this point, two groups of expression matrices were constructed from pre-processed datasets. The first group of expression matrices was obtained by merging all of the expression data from one species (see Fig. 1A). The GCNs that were constructed with these expression matrices were called GCNs based on multiple experiments (M-GCNs). The second group of expression matrices was constructed for each microarray experiment independently (see Fig. 1B). For each experiment, genes showing differential expression were identified and retained using the Significance Analysis of Microarrays (SAM) (Tusher, Tibshirani & Chu, 2001).

The GCNs that were constructed using this approach were called GCNs from single experiments (S-GCNs).

### Similarity measurement selection

A square similarity matrix (*S_nxn_*) was calculated for every single *E_nxp_*. The elements of *S_nxn_* or similarities (*s*_*i*,*j*_) between pairs of genes *i* and *j* were calculated using a similarity measure. We evaluated three similarity measures: the Absolute value of the Pearson Correlation Coefficient (APCC) (Zhang & Horvath, 2005), the Non-linear Correlation coefficient based on Mutual Information (NCMI) (Dionisio, Menezes & Mendes, 2004; Numata, Ebenhöh & Knapp, 2008) and the Normalized Mean Residue Similarity (NMRS) (Mahanta et al., 2012) (see Article S1, section 1). These measures were used to calculate the dependence between *X_i_* and *X_j_*, where *X_i_* is a continuous random variable denoting the expression level of the *i*th gene across samples (Meyer, Lafitte & Bontempi, 2008). These similarity measures take values in the same interval [0, 1], where 0 indicates non-dependence between *X_i_* and *X_j_*, and 1 indicates total dependence or maximum similarity. A detailed description of each similarity measure is given in the Article S1, section 1.

The *S_nxn_* were contrasted in dispersion plots. The similarity measurement that better detected not only the linear dependences between *X_i_* and *X_j_* but also the non-linear and scaled patterns was chosen.

### Similarity threshold selection

Once the *S_nxn_* was calculated using the chosen similarity measure, a similarity threshold τ^*^ was selected. The τ^*^ allowed us to determine the GCN edges according to the adjacency function given by Eq. (1) (Zhang & Horvath, 2005). Each GCN was represented by an adjacency matrix *A_nxn_* whose elements *a*_*i*,*j*_ take the value of 1 when the genes/nodes *i* and *j* are connected by an edge. We restricted the GCNs to have undirected edges and no self-loops; therefore, *A_nxn_* is symmetric with diagonal elements equal to 0. The GCNs were drawn using Cytoscape (Shannon et al., 2003).

(1) \begin{eqnarray*} {a}_{i,j}=\left\{\begin{array}{ll} \displaystyle 1&\displaystyle \text{if }{s}_{i,j}\geq {\tau }^{\ast }\\ \displaystyle 0&\displaystyle \text{if }{s}_{i,j}\lt {\tau }^{\ast }. \end{array}\right. \end{eqnarray*}

In this work, we followed an intuitive method based on the network’s topological properties for τ^*^ selection (Elo et al., 2007). The observed clustering coefficient in the GCN $C\left({\tau }_{v}\right)$ was compared with the expected clustering coefficient ${C}_{r}\left({\tau }_{v}\right)$ for a randomized GCN with the same degree distribution of the original GCN (Newman, 2003). Both clustering coefficients are contrasted as the similarity threshold increased (Eqs. (2) and (3)).

(2) \begin{eqnarray*} C\left({\tau }_{v}\right)=\frac{1}{K}\sum _{k_{i}\gt 1}\frac{2{D}_{i}}{{k}_{i}\left({k}_{i}-1\right)}. \end{eqnarray*}

In Eq. (2), the observed clustering coefficient $C\left({\tau }_{v}\right)$ is the average of the clustering coefficients of all the nodes in the GCN, so it could be also called “average clustering coefficient”; *k_i_* denotes the number of neighbors of gene *i* or node degree;  *D_i_* denotes the number of edges between the neighbors of gene *i*. *K* is the number of genes with *k_i_* > 1.

(3) \begin{eqnarray*} {C}_{r}\left({\tau }_{v}\right)=\frac{{\left(\overline{{k}_{d}}-\bar {k}\right)}^{2}}{{\bar {k}}^{3}N}. \end{eqnarray*}

In Eq. (3): *N* denotes the number of connected nodes in the GCN, $\bar {k}=1/N\sum _{i=1}^{N}{k}_{i}$, and $\overline{{k}_{d}}=1/N\sum _{i=1}^{N}k_{i}^{2}$.

According to Elo et al. (2007), the similarity threshold selection is determined by finding the minimum threshold *τ_v_* for which the difference between the clustering coefficients is maximum. Although this strategy is useful for a wide broad of networks, it is not suitable for those networks where $\left(C\left({\tau }_{v}\right)-{C}_{r}\left({\tau }_{v}\right)\right)\lt 0$. Here, we use the absolute difference between clustering coefficients (Eq. (4)). Thus, τ^*^ is the first local maximum of the curve $\vert C\left({\tau }_{v}\right)-{C}_{r}\left({\tau }_{v}\right)\vert $.

(4) \begin{eqnarray*} {\tau }^{\ast }={\mathrm{min}\atop v}\left\{{\tau }_{v}:\vert C\left({\tau }_{v}\right)-{C}_{r}\left({\tau }_{v}\right)\vert \gt \vert C\left({\tau }_{v+1}\right)-{C}_{r}\left({\tau }_{v+1}\right)\vert \right\}. \end{eqnarray*}

In Eq. (4), τ^*^ is the selected similarity threshold; *τ*_*v*+1_ = *τ_v_* + 0.01 with *τ_v_*∈[0.01,  0.99].

The validity of this modification was evaluated with simulated networks. The simulation procedure and results are described in Article S1, section 2; Fig. S3.

### GCN comparison by Principal Component Analysis (PCA)

The GCNs were characterized by eight graph variables (Fig. 1C). These informative measurements were selected following different requirements. Initially, we selected a subset of four variables that explain topological properties of reconstructed networks. For example, to study the structure of networks and their tendency to form sets of tightly connected edges, the clustering coefficient was used. Besides, the density of edges allowed us to measure whether the network is tight or cohesive (Horvath & Dong, 2008). To average the importance of nodes in terms of its centrality a measure of centralization was used. This measure assumes that the greater the number of paths in which a node participates, the higher the importance for the network (Costa et al., 2005). Equally, networks could show high variance in their nodes connectivity, especially in scale-free topologies. We assessed the heterogeneity measure to reveal whether the networks have heterogeneous connectivity (Horvath & Dong, 2008).

Subsequently, we planned to study the structure of networks adding external information. For this purpose, a subset of four variables was proposed as follows. Given that nodes in coexpression networks also represent coded proteins with different biological functions, it’s interesting to consider that nodes are not homogenous. To measure how much the nodes link to others with similar or dissimilar characteristics, a pair of assortativity coefficients was introduced. These coefficients merge current topological information with external Gene ontology (GO) annotations and PFAM annotations.

In the same way, we used graph theory to study the relationship between gene significance and connectivity. We assessed the correlation between node degree and presence of typical domains found in the immunity proteins. The correlation takes a reference dataset of genes encoding proteins involved in defense. We evaluated whether highly connected hub nodes are central to the network structure but also biologically significant in immune responses.

As this work focused on plant pathogen interactions, the tolerance to attacks as represented by the action of the effectors as suppressors of plant immunity was considered important. It was recently demonstrated that effector proteins from pathogens are directed to hubs of plant immunity networks (Mukhtar et al., 2011). Here, we analyzed the resistance to these perturbations by means of the average path length (Albert & Barabasi, 2002). A detailed description of these eight variables is annexed in the Article S1, section 3.

The M-GCNs and S-GCNs were compared in separated collections after characterization. Initially, the characterization matrices *T_gxt_* of *g* networks by *t* variables were formed for M-GCNs and S-GCNs. Subsequently, a PCA for every single *T_gxt_* was conducted (Jolliffe, 2002). Those principal components (PCs) retaining more variance were selected.

The M-GCNs and S-GCNs were analyzed using the PCs planes. In addition, two procedures were considered for S-GCNs comparison:

(i) We classified every S-GCN by the treatment studied in the experiment. In this work, the experiments included stresses caused not only by pathogens but also by chemical substances that are related to pathogen activity and the plant immune system. Those stresses sharing similar pathogens or chemical substances were grouped (Table 1). Subsequently, those S-GCNs belonging to the same stress group were depicted on the PCs planes.
(ii) The K-means algorithm was used to find clusters of S-GCNs on the PCs planes. We selected the optimum number of clusters based on the Bayesian Information Criterion (BIC). This selection was achieved using the R adegenet library (R Development Core Team, 2011). The clusters were analyzed with the stress groups as previously defined.

The R code for the construction and comparison of GCNs is given in the Script S1.

**Table 1:** Pathogen resistance microarray data collected.

Id.	GEO dataset	Plant	Stress group	Stress
1	GSE12856	*Arabidopsis*	PTI	Non-host
2	GSE13739	*Arabidopsis*	Induced resistance	Induced resistance (SA)
3	GSE14961	*Arabidopsis*	Induced resistance	Induced resistance (SA)
4	GSE15236	*Arabidopsis*	Fungi	*Fusarium oxysporum*
5	GSE16471	*Arabidopsis*	PTI	PTI
6	GSE16472	*Arabidopsis*	PTI	PTI
7	GSE16497	*Arabidopsis*	Induced resistance	Induced resistance (Aphid)
8	GSE17382	*Arabidopsis*	PTI	PTI
9	GSE17875	*Arabidopsis*	Fungi	*Botrytis cinerea*
10	GSE19273	*Arabidopsis*	Bacteria	*Ralstonia solanacearum*
11	GSE20188	*Arabidopsis*	Induced resistance	Induced resistance (insecticides)
12	GSE21762	*Arabidopsis*	Induced resistance	Induced resistance (JA)
13	GSE21920	*Arabidopsis*	Bacteria	*Pseudomonas syringae*
14	GSE26679	*Arabidopsis*	Fungi	*Golovinomyces cichoracearum*
15	GSE26973	*Arabidopsis*	Induced resistance	Induced resistance (exudates)
16	GSE28800	*Arabidopsis*	Induced resistance	Induced resistance (chemistry)
17	GSE431	*Arabidopsis*	Fungi	*Erysiphe cichoracearum*
18	GSE5513	*Arabidopsis*	Induced resistance	Induced resistance (PTI)
19	GSE5752	*Arabidopsis*	Induced resistance	Induced resistance (SA)
20	GSE5753	*Arabidopsis*	Induced resistance	Induced resistance (SA)
21	GSE5754	*Arabidopsis*	Induced resistance	Induced resistance (SA)
22	GSE5755	*Arabidopsis*	Induced resistance	Induced resistance (SA)
23	GSE5756	*Arabidopsis*	Induced resistance	Induced resistance (SA)
24	GSE5757	*Arabidopsis*	Induced resistance	Induced resistance (SA)
25	GSE5758	*Arabidopsis*	Induced resistance	Induced resistance (SA)
26	GSE6176A	*Arabidopsis*	PTI	PTI
27	GSE6176B	*Arabidopsis*	Bacteria	*Pseudomonas syringae*
28	GSE6831	*Arabidopsis*	Induced resistance	SAR (JA)
29	GSE8319	*Arabidopsis*	PTI	PTI
30	GSE10426	*Arabidopsis*	Fungi	*Plasmodiophora brassicae*
31	GSE10713	*Arabidopsis*	Fungi	*Fusarium oxysporum* pv. raphani
32	GSE13390	*Arabidopsis*	Bacteria	*Pseudomonas syringae* pv. tomato
33	GSE15880	*Arabidopsis*	Fungi	*Botrytis cinerea*
34	GSE15881	*Arabidopsis*	Fungi	*Botrytis cinerea*
35	GSE18757	*Arabidopsis*	Bacteria	*Ralstonia solanacearum*
36	GSE25838	*Arabidopsis*	Fungi	*Botrytis cinerea*
37	GSE34081	*Arabidopsis*	Bacteria	*Pseudomonas syringae* pv. tomato
38	GSE7990	*Arabidopsis*	Induced resistance	Induced resistance (ISR, *Bradyrhizobium*)
39	GSE8877	*Arabidopsis*	Fungi	*Plasmodiophora brassicae*
40	GSE31230	*Arabidopsis*	Bacteria	*Ralstonia solanacearum*
41	GSE19239	Rice	Bacteria	*Xanthomonas oryzae* pv. oryzicola
42	GSE32582	Rice	Oomycetes	*Pythium graminicola*
43	GSE33411	Rice	Bacteria	*Xanthomonas oryzae* pv. oryzae
44	GSE7256	Rice	Fungi	*Magnaporthe grisea*
45	GSE8216	Rice	Induced resistance	Induced resistance (cellulase)
46	GSE16470	Rice	Fungi	*Magnaporthe oryzae*
47	GSE28308	Rice	Fungi	*Magnaporthe oryzae*
48	GSE36093	Rice	Bacteria	*Xanthomonas oryzae* pv. oryzae
49	GSE29740A	Soybean	Fungi	*Phakopsora pachyrhizi*
50	GSE29740B	Soybean	Fungi	Soybean rust
51	GSE33410	Soybean	Fungi	Soybean rust
52	GSE8432	Soybean	Fungi	*Phakopsora pachyrhizi*
53	GSE9687	Soybean	Oomycetes	*Phytophthora sojae*
54	GSE21999	Tomato	Fungi	*Colletotrichum coccodes*
55	GSE14637	Tomato	Fungi	*Botrytis cinerea*
56	GSE33177	Tomato	Oomycetes	*Phytophthora infestans*
57–59	—	Yuca	Bacteria	*Xanthomonas axonopodis* pv. manihotis

## Results

With the aim of generating a general picture of the immunity networks, microarray data from different plants in response to pathogens were used to construct GCNs. The general methodology that was followed to construct and compare the GCNs involved four steps: (1) the construction of expression matrices, (2) the selection of a similarity measurement and the construction of gene similarity matrices, (3) the similarity threshold selection and (4) the comparison of GCNs (Fig. 1).

### Expression matrices construction

A total of 59 raw microarray datasets from pathogen-infected plants were obtained from publicly available data that were pre-processed and filtered (see Methods and Table 1). *Arabidopsis* and rice were represented by more experiments than were the other species; 40 and 8 experiments, respectively. In *Arabidopsis*, studies with the pathogens *Botrytis cinerea* and *Pseudomonas syringae* pv. tomato were the most abundant. For rice, experiments involving *Magnaporthe oryzae* and *Xanthomonas oryzae* pv. oryzae were the most common. Soybean, tomato and cassava are less studied plants and, therefore, the number of experiments using these species was scarce. A total of 5, 3 and 3 experiments, respectively, involving these species were used.

Two groups of expression matrices were constructed from pre-processed datasets. The expression matrices used to construct the M-GCNs are summarized in Table 2. The expression matrices used to construct the S-GCNs are summarized in Table S1. As expected, the number of samples and genes in the expression matrices was higher for plants with more experiments (*Arabidopsis* and rice).

**Table 2:** Main results for M-GCN construction: expression matrices dimensions, similarity thresholds and network sizes.

Plant	Expression matrix		Similarity threshold	M-GCN size	
	Samples	Genes		Nodes	Edges
*Arabidopsis*	560	21,122	0.91	1,563	4,489
Rice	136	32,475	0.89	744	3,065
Soybean	385	13,853	0.92	762	6,356
Tomato	33	7,405	0.92	674	5,794
Cassava	87	3,736	0.94	307	739

### Similarity measurement selection and construction of similarity matrices

Three similarity measurements were evaluated to assess the similarity matrix between genes. We compared the dispersion plots of the similarities that were calculated using the APCC (${s}_{i,j}^{A P C C}$), NCMI (${s}_{i,j}^{N C M I}$) and NMRS (${s}_{i,j}^{N M R S}$); formally ${s}_{i,j}^{A P C C}$ vs. ${s}_{i,j}^{N C M I}$ and ${s}_{i,j}^{A P C C}$ vs. ${s}_{i,j}^{N M R S}$ (Fig. S1).

For low ${s}_{i,j}^{A P C C}$ in which no linear similarity is detected, the high values of ${s}_{i,j}^{N C M I}$ and ${s}_{i,j}^{N M R S}$ evidence a nonlinear correlation (Fig. S1). In other words, for low Pearson coefficients in which no linear similarity is detected, the NCMI and NMRS were able to detect nonlinear correlation. The genes with linearly correlated expression profiles are placed in the upper right corner, and the genes with nonlinearly correlated expression profiles can be found in the upper left corner. Based on these comparisons, we concluded that NMRS and NCMI are both useful measures in detecting linear and non-linear correlations. Nevertheless, non-linear correlations were better revealed by NCMI. This result is especially important when a similarity threshold τ^*^ is chosen based on the gene pairwise similarity matrix, because some gene pairs with a non-linear correlation would be included in the final gene network. Moreover, for any τ^*^ > 0.5, the number of edges from the non-linearly correlated profiles will be greater if ${s}_{i,j}^{N C M I}$ is used (Fig. S1). Given that our goal was to construct GCNs including linear and non-linear relationships between genes, we decided that NCMI was the best metric among the three approaches that were evaluated.

### Similarity threshold selection and GCN construction

**Figure 2:**
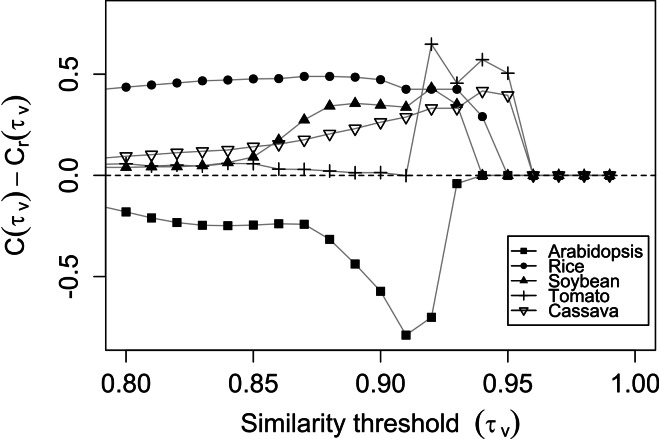
The application of methodology for the similarity threshold selection in the M-GCN. The differences between the observed clustering coefficients *C*(*τ_v_*) and the expected clustering coefficients for a randomized network *Cr*(*τ_v_*) are shown when the similarity threshold (*τ_v_*) is increased.

The similarity matrices were used to test the methodology for the threshold selection. In the M-GCN construction, Fig. 2 shows the difference between the expected clustering coefficient of the random network ${C}_{r}\left({\tau }_{v}\right)$ (Elo et al., 2007) and the real clustering coefficient that was based on the constructed network $C\left({\tau }_{v}\right)$ (see Methods). The curves show a first phase of continuous growth where the non-significant edges are gradually removed (Fig. 2). The maximum difference is reached when well-defined clusters are formed due to the removal of non-relevant edges. The clustering coefficient of the random network should remain lower than that of the real network, as assumed by Elo et al. (2007); however, the curve of *Arabidopsis* did not show the expected behavior.

The *Arabidopsis* curve (Fig. 2) showed that the methodology proposed by Elo et al. (2007) is not suitable for networks where $\left(C\left({\tau }_{v}\right)-{C}_{r}\left({\tau }_{v}\right)\right)\lt 0$, indicating that ${C}_{r}\left({\tau }_{v}\right)\gt C\left({\tau }_{v}\right)$. In this work, a minor adaptation of the method was proposed (see Eq. (4) in Methods). Indeed, several alternative ways to utilize the clustering coefficient in the threshold selection can be studied (Elo et al., 2007) and the global optimization problem expressed in Eq. (4) is not unique. Through simulation we determined that the absolute value of the differences between $C\left({\tau }_{v}\right)$ and ${C}_{r}\left({\tau }_{v}\right)$ is suitable for the threshold selection. Accordingly, the maximum absolute value between clustering coefficients is still a reference point to identify the transition between the underlying biological system and those random relationships embedded in the similarity matrix. The adaptation relies also in the basis that the maximum the absolute value, the maximum the difference between real and randomized systems. We successfully applied this adaptation for the entire threshold selections performed in our work.

**Figure 3:**
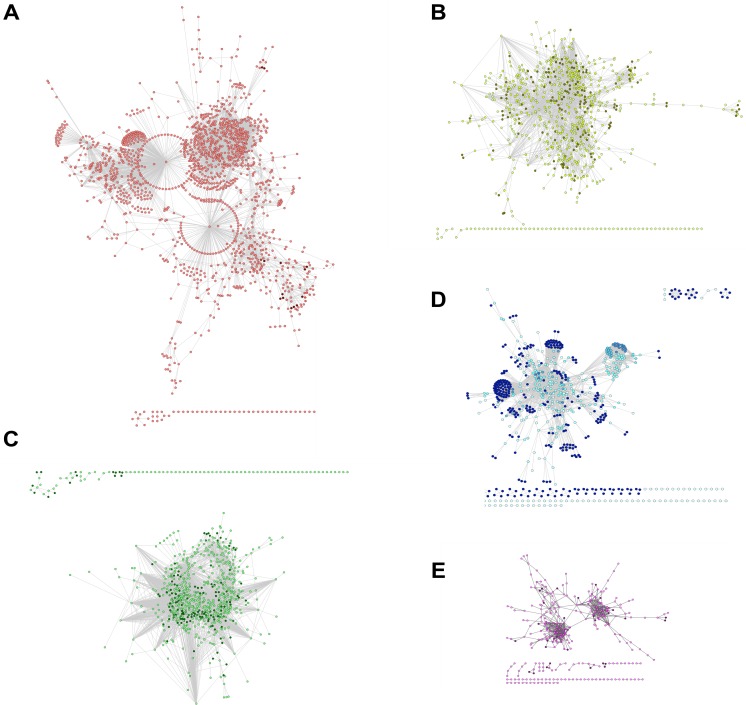
The M-GCNs for the five plants. (A) *Arabidopsis*, (B) rice, (C) soybean, (D) tomato and (E) cassava. The nodes that have high clustering coefficients are mapped to dark colors.

The similarity threshold that was obtained for the *Arabidopsis* M-GCN was the lowest (0.89), and its network was the largest among the five plants (Table 2; Fig. 3). The thresholds for the S-GCNs had a wide range of values (0.27–0.93) for all of the species (Table S1). The largest S-GCNs (ids: 8, 44, 6, 13, 40) had more than 1,500 nodes and belonged to experiments that used *Arabidopsis* and rice.

From Table 2 and Fig. 3 we inferred that the species with more expression data or experiments have larger M-GCNs. Indeed, an association between the number of nodes and the number of samples in the expression matrix was found: PCC = 0.98 (*p*-value = 0.002). Consequently, the size of the M-GCNs is due to the inclusion of very diverse experiments. When a greater number of different types of experiments are included in the expression matrix, the number of nodes/genes required to represent the underlying immunity system is higher. This requirement is because more information about several functions is presented as different experiments are used.

For the S-GCNs, however, we did not found a clear relationship between the quantity of expression data and the network size. The correlation between the number of nodes and the number of samples from each S-GCN is very low: PCC = −0.24 (*p*-value = 0.004). In other words, although the size of the S-GCNs is highly variable, this variation is neither correlated with the number of experimental data points nor dependent on the organism.

### Comparison of GCNs by Principal Component Analysis (PCA)

For these analyses, we focused on the two groups of GCNs, 59 S-GCNs (summarized in Table S1) and 5 M-GCNs (summarized in Table 2). We aimed to compare the obtained networks between species and experiments. The networks were characterized by eight graph variables: (1) the clustering coefficient, (2) the centralization, (3) the coefficient of variation of the node degree (also known as heterogeneity), (4) the network density, (5)–(6) assortativity coefficients, (7) the tolerance to attacks and (8) the correlation between the node degree and the presence of immunity domains (see Methods).

**Figure 4:**
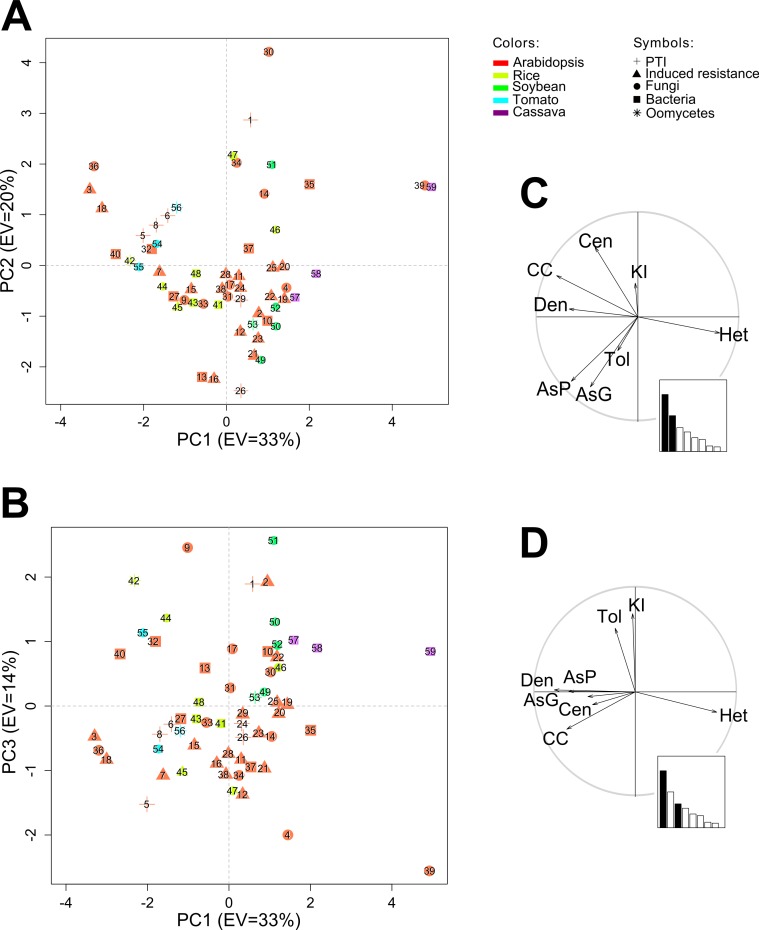
The differentiation of the S-GCNs using the PCA. (A), (B) The projection of the S-GCNs in the planes (A) PC1–PC2 and (B) PC1–PC3. The networks were numbered with the same ids. from Table 1 and are represented with symbols according to the stress group. The axes labels show the percentage of the explained variance (EV) by each principal component. (C), (D) The correlation circles for the variables in the planes (C) PC1–PC2 and (D) PC1–PC3. The bar plot consists of Eigenvalues. The variables are represented with labels: the clustering coefficient (CC), the centralization (Cen), the heterogeneity (Het), the density (Den), the assortativity coefficient from the GO (AsG), the assortativity coefficient from the PFAM (AsP), the tolerance to attacks (Tol) and the correlation between the node degree and the presence of immunity domains (KI).

The characterization matrices for the S-GCNs and M-GCNs were constructed with these variables (Tables S1 and S2). These variables were then summarized using the PCA. The S-GCNs and M-GCNs were projected in the principal component (PC) space (Fig. 4; Fig. S2).

#### Analysis of PCs used to project S-GCNs

The first three PCs were selected and used to represent the data structure in 2D plots (Fig. 4). PC1, PC2 and PC3 explain 33%, 20% and 14% of the total variance, respectively. Accordingly, 67% of the total information is represented in these plots. The PC1 (33%) explains primarily the information that is contained in the variables of heterogeneity and density, the clustering coefficient and the assortativity coefficient (PFAM), predominantly topological information (see Fig. 4C; Table S3 shows each variable’s contribution to the principal components). The PC2 (20%) explains the assortativity coefficient and the centralization, primarily non-topological information. The PC3 (14%) explains the tolerance to attacks and the dependence between node degree and immunity domains (see Fig. 4D). These last variables were not explained by PC1 or PC2; consequently, PC3 is associated mainly with the robustness of the immunity processes.

The dependence of the graph variables with the network size was also studied to verify that characterization of networks was not affected by their size. The PCC between the number of nodes and the graph variables clearly shows that all of the variables exhibited a very small correlation with the size of the network (Table S3); this assures that the PCA was not affected or biased by differences in the S-GCN sizes.

#### Differentiation of S-GCNs between species

The PCA plots allowed us to differentiate S-GCNs among species. The *Arabidopsis* S-GCNs are spread over the planes PC1–PC2 and PC1–PC3 (Figs. 4A and 4B). Due to this dispersion, we deduced that *Arabidopsis* S-GCNs have very different graph variables depending on the experiment analyzed.

In contrast, S-GCNs from other plants were more similar based on the eight variables and, therefore, clustered into specific zones (Figs. 4A and 4B). For example, there was a clear difference between cassava and tomato S-GCNs on PC1. Tomato S-GCNs are denser and more clustered than cassava S-GCNs. Cassava S-GCNs have high heterogeneity. Furthermore, the cassava and soybean S-GCNs were significantly more tolerant to attacks than those of the other species.

Another example of differentiation among species was found in rice. There is a defined group of 5 rice S-GCNs near to the center of the PC1–PC2 plane (Fig. 4A). Their assortativity coefficients are slightly higher than other S-GCNs, indicating that co-expressed genes in rice networks shared more functional annotations than did genes from other plants. These examples demonstrate that variables used for the characterization were useful in differentiating S-GCNs among species. In Article S1, section 4, we explain the position of S-GCNs by the contribution of each variable to the PCs.

#### Differentiation of S-GCNs between stress groups

The PCA plots allowed us to find similar S-GCNs based on stress groups. A total of five stress groups were defined: Bacteria, fungi, induced resistance, oomycetes and PTI (see Table 1). These stress groups are highlighted using different symbols in Fig. 4.

Networks that were constructed under conditions from the same stress group were found close to each other. For instance, we found that networks 27, 41, 43 and 48 are close to each other and no separation is observed in both planes (Fig. 4). These networks are associated with studies of bacteria in *Arabidopsis* (id 27; *Pseudomonas syringae* pv. tomato) and rice (ids 41, 43, 48; *Xanthomonas oryzae* pv. oryzae and *Xanthomonas oryzae* pv. oryzicola). In this way, they showed similar graph variables but also could represent comparable immunity process against bacteria in these two species.

Some S-GCNs sharing similar stress groups were also identified in quadrant I of the PC1–PC2 plane (Fig. 4A). For example, networks 34 and 47, which are related to fungi experiments in *Arabidopsis* (ids 34; *Botrytis cinerea*) and rice (id 47; *Magnaporthe oryzae*). In the PC1–PC2 plane, they are forming a closer pair; therefore, their topological variables (clustering coefficient, density, heterogeneity and centralization) are analogous. Because of their position in PC2, we can conclude that they are disassortative and their linked genes do not share many functional annotations. Both networks are also close in the PC1–PC3 plane. Therefore, we can infer that the immunity processes that are represented in these networks (derived from plant–pathogen interactions of rice-*Magnaporthe oryzae* and *Arabidopsis*-*Botrytis cinerea*) could share some similarities.

Despite the previous examples, some networks from the same group of stresses were also found separated. An example of opposing S-GCNs is the pair of networks 9–39. They are related to fungal (*Botrytis cinerea*, *Plasmodiophora brassicae*) experiments in *Arabidopsis*. Both networks are in total opposition in the three PCs. While network 9 is robust and assortative, network 39 is less tolerant to attacks and shows high heterogeneity. A similar result was observed for *Arabidopsis* networks 10, 35 and 45 from *Ralstonia solanacearum*. Consequently, even when two networks are associated with the same stress or group of stresses, their graph variables could differ.

#### Clustering of S-GCNs using the K-means algorithm

**Figure 5:**
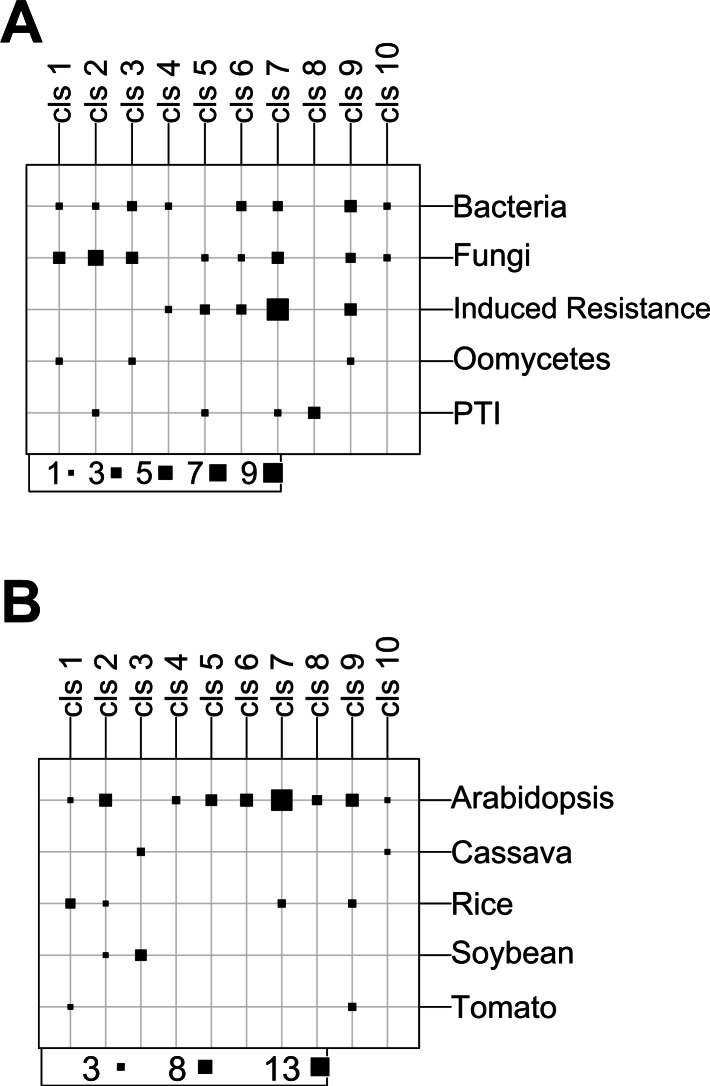
The results of the S-GCN clustering using the K-means algorithm. A graphic view of the cross-Tables comparing the clusters (cls) and (A) stress groups or (B) species. The square size increases with the number of S-GCNs.

The K-means algorithm was used with the aim of finding clusters of S-GCNs (see Methods). We selected an optimum of 10 clusters (Fig. S4). Mainly, induced resistance experiments were gathered together in cluster 7, and PTI stresses were in cluster 8 (Fig. 5). Bacteria and fungi were present in almost all of the clusters.

Possible associations between clusters and stress groups were revealed (Fig. 5A; Table S1). For example, cluster 9 grouped some *Arabidopsis*, rice and tomato networks. In this cluster, networks 36 (*Botrytis cinerea*) and 54 (*Colletotrichum coccodes*) share the same stress group (Fungi). Networks 27 and 32 (*Pseudomonas syringae* pv. tomato) were both from bacteria stresses. Also, networks 7, 18 and 45 were related to induced resistance experiments. This result shows that, for specific networks, a small distance in the PC space could have a biological meaning in correspondence with the experiment.

Besides, experiments related to PTI and salicylic acid (SA) were grouped together (Table S1). For instance, in cluster 5, we found that network 26 from PTI was grouped with network 21 from SA. In cluster 7, network 29 from PTI was grouped with networks 19, 20, 23, 24 and 25 from SA. These findings implied that some stress groups, such as PTI and the induced resistance by SA, are potentially related to similar co-expression behaviors.

As expected, some clusters are enriched with S-GCNs from specific species (Fig. 5B). For instance, cluster 3 is useful to compare experiments from soybean and cassava. While clusters 4, 5, 6 and 8 are exclusively conformed by *Arabidopsis* networks. Accordingly, the clustering of S-GCNs with the K-means algorithm allowed a straightforward identification of theoretically similar networks based on topological and biological characteristics.

#### Comparison of M-GCNs

In relation to the M-GCNs comparison, two PCs were analyzed (Fig. S2). We verified that networks with low clustering coefficients had high heterogeneity. Both assortativity coefficients showed information that was different from that of the topological variables, such as the density and clustering coefficient.

From the PCA plot, we conclude that *Arabidopsis* M-GCN constitute a network with high heterogeneity, but is also more tolerable to attacks. Cassava M-GCN is a disassortative and non-centralized network, and rice, tomato and soybean M-GCNs constitute highly clustered and dense networks.

## Discussion

With the aim of obtaining a general representation of the events that are triggered during plant immune responses and to compare these responses in different plants against diverse pathogens or pathogen response stimuli, GCNs were constructed from the available microarray data from *Arabidopsis*, rice, soybean, tomato and cassava. A careful selection of the methodology at each step was undertaken to fulfill two main criteria: enhanced objectiveness and enhanced information extraction from the gene expression data.

The careful analyses of the linear and non-linear relationships between gene expression profiles allowed us to select NCMI as the best metric approach. Then, the similarity thresholds were defined by the clustering coefficient method. The GCNs were obtained for the different plants in response to different stimuli. Networks were characterized by graph variables and a PCA was applied. Each network showed a specific pattern and topology, indicating that the networks are species-specific, dynamic entities, and even for the same species in response to the same pathogen, the networks can be quite different (Fig. 4). The comparative GCN analyses between species allowed for the identification of some common elements, indicating a cross-talk between the different signaling responses to pathogens (Fig. 5).

We investigated different factors that should be considered when GCNs are used to propose biological hypotheses. For some plant species, both the number of experiments and the completeness of the genome annotations were inadequate. In some cases, expression data were missing for several genes. These factors reduced the data representativeness, especially for tomato, cassava and soybean, for which expression data were not available for all of the genes of the genome. We observed that the genes in the expression matrices from these plants were incomplete, considering the number of genes that were reported in their genomes (Table 2). The microarray data for *Arabidopsis* and rice were of better quality, and the expression matrices contained information for almost all of the known genes. These differences in data availability were reflected in the final GCNs in the sense that the information represented in the networks from the plants with less data was also sparse.

Regardless of differences in the quantity and quality of the data, the experiments covered a broad spectrum of conditions. We considered experiments using plants inoculated with bacteria, fungi and oomycetes, including ETI and PTI responses and induced resistance experiments. This choice of experiments allowed for the gathering of a broad representation of immunity processes. Fifty-nine experiments offered a good balance between the representation of plant immunity processes and a sufficient number of samples for statistical analyses (Steuer et al., 2002).

Our methodology aims to have a simple application, low-level computational resources and accurate results to be easily implemented. This methodology for the construction of GCNs falls in a group of methodologies that are usually termed Relevance Networks based on their pairwise measures of similarity (Butte & Kohane, 2000). Evidently, more elaborate strategies involving further mathematical and statistical complexities at each step can be studied (López-Kleine, Leal & López, 2013); however, our interest was neither to study the molecular mechanisms in detail nor causal regulatory relationships among gene products. In this sense, at each step of the methodology, we objectively chose the best method from several available options. We recommend the following methods:

(1) NCMI as the similarity measurement: although the NCMI estimation was more complex than that of the APCC or NMRS, its advantages included the detection of non-linearly correlated pairs of genes and flexibility in detecting any type of dependence between expression profiles.
(2) The threshold definition based on the modified clustering coefficient method: among the methods proposed to objectively select a threshold, we used a method based on the topological features of graphs (Elo et al., 2007) that is easy to implement and is based on a simpler mathematical background (Luo et al., 2007). The method was slightly adapted to consider networks with high heterogeneity, as was the case for the *Arabidopsis* M-GCN.
(3) The characterization and comparison of GCNs using a PCA: the network comparison based on the topological variables such as density, heterogeneity or centrality allowed for the discovery of only similar patterns of morphology between GCNs. We added new non-topological variables to characterize the GCNs, including tolerance to pathogen attacks, assortativity coefficients related to functional annotations and dependence between node degree and immunity domains. These variables produced a better differentiation of GCNs in the PCs space and revealed biological conclusions about the co-expression systems studied.

The characterization of GCNs depends on the use of variables able to extract the most relevant features. There is an unlimited set of variables that could be selected to characterize networks (Costa et al., 2005). Thus, the inclusion or exclusion of variables relies on the knowledge of the problem. Here, we aimed to compare global patterns of immune responses reflected in coexpression networks. We included a set of variables that mutually exposed the differences among the studied phenomena and extract as much information as possible. However, we found that variables like the density and clustering coefficient were highly correlated, implying redundancy (Figs. 4C and 4D). Similarly, both assortativity coefficients contained equivalent information. We could expect that results will not be drastically altered after removing some of these variables. The clustering coefficient and the assortativity coefficient from GO could summarize adequately the variability observed in their counterparts. Alternatively, removing non-correlated variables could obscure the variability observed and results will change. For example, excluding the tolerance to attacks will reduce the differences between soybean S-GCNs and those of the other species (Fig. 4B). Likewise, adding new variables could reveal relationships not presented in our plots. As expressed by Costa et al. (2005), before altering the characterization matrix, it is of importance to have a good knowledge not only of the most useful variables, but also of their properties and interpretation.

The confidence in the constructed S-GCNs allowed for us to analyze the networks that were obtained for extracting biological knowledge and especially for comparing behaviors between and within species. As stated before, most of the experiments that were analyzed in this study were from *Arabidopsis*. A broad spectrum of gene expression data for this model plant is available (Schenk et al., 2000; Tao et al., 2003; Zipfel, 2009). The zigzag model that was developed to explain the evolution of plant immunity was constructed based on the knowledge of the pathosystem *Arabidopsis*-Pseudomonas (Jones & Dangl, 2006; Nishimura & Dangl, 2010). In this sense, the S-GCNs that were constructed during the SAR response or that were induced by SA were based on *Arabidopsis* data; these and other experiments have contributed significantly to a major understanding of this phenomenon (Schenk et al., 2000), including the identification and action mode of NPR1 and the WRKY transcription factors (Wang, Amornsiripanitch & Dong, 2006; Dempsey & Klessig, 2012).

We compared S-GCNs that were obtained from a deeply studied plant such as *Arabidopsis* with S-GCNs that were obtained from an almost unstudied plant with scarce transcriptomic data such as cassava. The S-GCNs comparison between these two plants showed that there are few common elements and that their topologies are different. However, the K-means allowed us to obtain a cluster that grouped *Arabidopsis* and cassava networks (cluster 10). This result is important because, for some genes with unknown functions in cassava, a role in immunity processes could be assigned based on these networks. Several studies have reported the utility of this strategy in assigning a putative function to unknown genes (Ficklin & Feltus, 2011; Hwang et al., 2011). Further experiments employing mutant versions of these genes and using silencing approaches will help to determinate the function of these genes in plant immunity.

We observed that the S-GCNs that were generated from *Arabidopsis*-*Pseudomonas syringae* pv. tomato DC3000 (PstDC3000) were very distant, even when they came from the same pathosystem (ids. 13, 27, 32, 37). However, even though these experiments belonged to the same plant–pathogen interaction (*Arabidopsis*-*Pseudomonas*), some of them used pathogens (ids. 13, 27 and 32) or plants (ids. 13 and 37) exhibiting mutations in particular genes. Furthermore, the samples were taken at different time-points in all of the experiments (see link to summary of experiments in Table 1). Taking together, these results suggest that minor changes, such as the mutation of individual genes in the plant or the pathogen, produce networks with different topologies. In addition, networks seem very dynamic given the important changes they suffer considering different time-points during the immune responses. This aspect indicates that the construction of a network represents only a reduced aspect of the whole gene co-expression in the cell at a given moment, and no generalization can be made for the entire life cycle of a plant cell.

The PTI and ETI responses shared similar responses (ion fluxes, production of ROS and activation of Map kinases); we expected to observe more similarities for the PTI and ETI networks. However, we observed that several PTI networks (ids. 5, 6, 8) were not similar to ETI GCNs, due to the highly dynamic nature of these cellular responses. Similar results were obtained experimentally, where the expression of only a few genes showed an overlap between the PTI and ETI (Navarro et al., 2004).

On the other hand, we observed that different networks that were constructed from experiments involving the PTI were very similar to each other, even when they correspond to induction for different MAMPs. For example, networks 5 and 29 are closer in PC2 and exemplify the induction of different MAMPs: flg22 and chitin. Previous studies have reported a very similar response to flagellin and Elongation Factor Tu (Zipfel, 2009). A similar situation was observed with networks that were constructed from induced resistance and that were grouped together (cluster 7). This result suggests that the PTI and induced responses are robust and are not strongly influenced by other environmental conditions. These types of robust responses were previously reported for incompatible interactions (Tao et al., 2003).

It is also interesting to note that the GCNs that were obtained from the PTI and induced responses were also similar (clusters 7 and 5), supporting previous experimental studies (Tsuda et al., 2008). The ETI has been considered a stronger but very specific response for a particular race of pathogens (Jones & Dangl, 2006). The distal-induced resistance that is activated once the ETI has started or the response induced by hormones such as SA also produces a weak but efficient response against a broad spectrum of pathogens. The PTI is weak as well but can confer resistance to a larger group of non-adapted pathogens. It would be interesting to study more in detail whether there is a relationship between a robust, weak response and the spectrum of resistance.

The rice networks in response to two different bacteria (*X. oryzae* pv. oryzae and *X. oryzae* pv. oryzicola) showed a high degree of similarity (ids. 41, 43, 48, Fig. 4). This result is interesting given that the two bacteria employ different strategies of infection. The first bacteria colonize the vascular system, and the others reside on the apoplast. Consequently, both bacteria produce different symptoms (Hajri et al., 2012). The similar network topologies that were observed in our study suggest that, although the colonization is different, the molecular plant responses and genes involved are related in both cases.

Another example comprises the network 46. This network was obtained from rice plants that were inoculated with *X. oryzae* pv. oryzae, but also shows some degree of similarity with a network from *Magnaporthe oryzae* (id. 41). Some of the pathways can be shared in response to different pathogens at particular times during the infection or response. Consequently, the networks can exhibit this type of similarity.

In response to similar pathogens, plants can activate conserved signaling pathways. For example, we observed that two unrelated plants such as *Arabidopsis* and rice (dicotyledonous and monocotyledonous) react in similar ways in response to bacteria (ids. 27, 41, 43, 48, Fig. 4). This response does not indicate that the genes are the same, but rather that some degree of conservation of their function exists. Therefore, it is possible that some plant responses to a particular group of pathogens can be more “stable” and conserved. Considering all of these observations, it is important to consider aspects such as the type of interaction (compatible, incompatible, non-host) evolutionary relationship and mode of colonization between pathogens, as well as the time-points after pathogen inoculation when identifying common or shared elements between the networks.

The networks that were constructed for a species by merging several experiments are different from each other. They have also different characteristics from the networks that were constructed from only one microarray experiment. Differences between S-GCNs and M-GCNs are especially striking for *Arabidopsis*, which questions the validity of the global network merging all of the experiments. Our results indicate that a global immunity process gene co-expression network is very difficult to construct and could hardly resume global information on this complex process. Moreover, the high level of diversity found between S-GCNs indicates that, depending on the pathogen and type of immunity process that is triggered, the obtained network will be different. Therefore, we conclude that global networks such as those that were previously constructed by Atias, Chor & Chamovitz (2009), Pop et al. (2010) and Mutwil et al. (2011) could mask important gene relationships that are characteristic of a particular process. Also, these global networks could enhance relationships that are specific to only one biological process. Those gene relationships that arise only under special environmental and biological circumstances are better represented by process-oriented networks such as those that were previously constructed by Nakashima, Ito & Yamaguchi-Shinozaki (2009) and Lee et al. (2011).

## Conclusions

As a major finding, the closeness of GCNs on the principal component space is indicative of similar plant immune responses and conserved signaling pathways. The comparison of GCNs suggests cross-talk between the different responses to pathogens within plant species. It is possible that some plant responses to a particular group of pathogens are not only conserved but also more robust. Theses similarities between S-GCNs are a valuable source of predictions that can be considered in future works.

The representation of coordinated transcription through GCNs is necessary to gain comprehensible knowledge from the underlying transcriptomes. We showed that global immunity process should not be explored using the M-GCN approach. The comparative S-GCNs analyses allowed to conclude that dynamic of molecular plant responses produce networks with different characteristics. As a consequence, M-GCNs cannot properly summarize the experimental data.

Neither a high level of computational resources nor intricate algorithms were used. Thus, methods from this work are still applicable to expression data that are generated by any biological processes. Our strategy to extract relevant information from networks provides a shortcut to advanced studies in translational functional genomics, assuring that current biological knowledge for model organisms and less studied species is analyzed in conjunction.

## Supplemental Information

10.7717/peerj.610/supp-1 Article S1 Supplemental Material

10.7717/peerj.610/supp-2 Table S1 The main results for S-GCNs construction The expression matrices dimensions, similarity thresholds, characterization variables and clusters where networks were grouped.

10.7717/peerj.610/supp-3 Table S2 The characterization matrix for M-GCNs

10.7717/peerj.610/supp-4 Table S3 The explained variance by each principal component

10.7717/peerj.610/supp-5Figure S1The evaluation of similarity measuresThe dispersion plots of similarities were calculated using the *Arabidopsis* gene expression profiles: (A) The pair-wise comparison of ${s}_{i,j}^{A P C C}$ and ${s}_{i,j}^{N C M I}$ (B) The pair-wise comparison of ${s}_{i,j}^{A P C C}$ and ${s}_{i,j}^{N M R S}$.

10.7717/peerj.610/supp-6 Figure S2 The differentiation of the M-GCNs using PCA (A) The projection of M-GCNs in the plane PC1–PC2. The axes labels show the percentage of the explained variance (EV) by each principal component. (B) The correlation circles for the variables in the planes. The bar plot consists of Eigenvalues. The variables are represented with labels: the clustering coefficient (CC), the centralization (Cen), the heterogeneity (Het), the density (Den), the assortativity coefficient from the GO (AsG), the assortativity coefficient from the PFAM (AsP), the tolerance to attacks (Tol) and the correlation between the node degree and the presence of immunity domains (KI).

10.7717/peerj.610/supp-7 Figure S3 The dispersion of the calculated thresholds and theoretical thresholds for three groups simulated networks (SNs) The absolute error (**η**) is given for each group. (A) Group 1: The SNs with the same properties as the *Arabidopsis* M-GCN (*CV*(*k*) = 2.86, *C*(*t_v_*) = 0.02 and degree distribution *P*(*k*) *k*^−2^). (B) Group 2: The SNs with *CV*(*k*) = 1.00 and the same *C*(*t_v_*) and degree distribution as the *Arabidopsis* M-GCN. (C) Group 3: The SNs with *CV*(*k*) = 3.10 and the same *C*(*t_v_*) and degree distribution as the *Arabidopsis* M-GCN.

10.7717/peerj.610/supp-8 Figure S4 Bayesian Information Criterion (BIC) evolution when the number of clusters is increased An optimum of 10 cluster was selected (BIC = 107) using the elbow method heuristic.

10.7717/peerj.610/supp-9 Script S1 The R code for the construction and comparison of GCNs
