# Supplementary material for: Construction and comparison of gene co-expression networks shows complex plant immune responses
Source: PeerJ. 2014 Oct 9;2:e610. doi: 10.7717/peerj.610 (PMC4194462; doi:10.7717/peerj.610)
Supplement: Article S1 [file peerj-02-610-s001.pdf]

Supplemental Material

Article title: Construction and comparison of gene co-expression networks shows complex plant immune responses

Author names and affiliation:

Luis Guillermo Leal[1*], Email: lgleala@unal.edu.co

Camilo López[2], Email: celopezc@unal.edu.co

Liliana López-Kleine[1], Email: llopezk@unal.edu.co

[1] Department of Statistics, Universidad Nacional de Colombia, Bogotá, Colombia. Postal Code: 111321
[2] Department of Biology, Universidad Nacional de Colombia, Bogotá, Colombia. Postal Code: 111321

1. Similarity measures

1.1 Absolute value of the Pearson Correlation Coefficient (APCC)

The Pearson Correlation Coefficient (PCC) is defined by (Soranzo et al. 2007):

$$\rho(X_i, X_j) = \frac{\sum_{l=1}^{p}(x_i(l) - \overline{x_l})(x_j(l) - \overline{x_j})}{\sqrt{\sum_{l=1}^{p}(x_i(l) - \overline{x_l})^2 \sum_{l=1}^{p}(x_j(l) - \overline{x_j})^2}} \ [S.1]$$

In equation S.1, $\rho(X_i,X_j)$ denotes the PCC. $x_i(l)$ denotes the expression values of gen *i* across *l=1,…,p* samples and $\overline{x_l}$ denotes the mean of expression.

In this work, the absolute value of the PCC was used as similarity measure (Zhang and Horvath 2005):

$$s_{i,j}^{APCC} = |\rho(X_i, X_j)| \ [S.2]$$

1.1.1 Non-linear Correlation coefficient based on Mutual Information (NCMI)

In order to calculate the Mutual information (MI), an estimation of entropy for discrete random variables is required (Daub et al. 2004). Equation S.3 describes MI in terms of entropy and equation S.4 describes the entropy of a random variable in terms of a probability measure:

$$I(X_i, X_j) = H(X_i) + H(X_j) - H(X_i, X_j) \ [S.3]$$

$$H(X_i) = -\sum_{k_i \in B_i} p_{X_i}(x_{k_i}) \, log\left(p_{X_i}(x_{k_i})\right) \quad [S.4]$$

In equation S.4, $I(X_i,X_j)$ denotes the MI between variables. $H(X_i)$ denotes the entropy of a random variable X_i and $p(x_i)$ its probability measure. k_i denotes the discrete random variable's bin or subinterval. B_i denotes the bin index vector denoting the bin's number where $p(x_{ki})$ is estimated.

In this work, the random variables were categorized in n_b bins using the Global Equal Width algorithm (Meyer et al. 2008), where n_b was assessed by the Sturges' rule: $n_b = 1+\log_2(p)$ (Scott and Sain 2005). To estimate the entropy, the estimator proposed by Hausser (2006) was selected. MI was calculated using the R (R Development Core Team 2011) minet library (Meyer et al. 2008).

As MI takes values in the interval [0,∞), a transformation was applied to find the NCMI which falls into the range [0,1] (Dionisio et al. 2004):

$$s_{i,j}^{NCMI} = \sqrt{1 - e^{-2I(X_i,X_j)}} \quad [S.5]$$

1.1.2 Normalized Mean Residue Similarity (NMRS)

This metric is defined by (Mahanta et al. 2012):

$$s_{i,j}^{NMRS} = 1 - \frac{\sum_{l=1}^{p} \left| x_i(l) - \overline{x_i} - x_j(l) + \overline{x_j} \right|}{2 \cdot max\left\{ \sum_{l=1}^{p} \left| x_i(l) - \overline{x_i} \right|, \sum_{l=1}^{p} \left| x_j(l) - \overline{x_j} \right| \right\}} \quad [S.6]$$

In equation S.6, $x_i(l)$ denotes the expression values of gen *i* across *l=1,…,p* samples. $\mathbf{x}_i$ and σ_i denotes the mean and the variance of expression.

2. The method for threshold similarity selection

The *Arabidopsis* curve in Fig. 2 indicated that the methodology proposed by Elo et al. (2007) is not suitable for networks where the expected clustering coefficient of the random network $C_r(\tau_v)$ is higher than the clustering coefficient based on the constructed network $C(\tau_v)$. An adaptation of the method used for the threshold similarity selection was proposed (see equation 4).

2.1 The algorithm to simulate GCNs

The steps to evaluate the accuracy of equation 4 for similarity threshold selection were the following:

i) The M-GCN for Arabidopsis was constructed using the threshold given by equation 4. The coefficient of variation of node degree $CV(k)$, the clustering coefficient $C(\tau_v)$ and the degree distribution $(P(k) \sim k^{\gamma})$ from Arabidopsis M-GCN were obtained.

ii) Three groups of 100 SNs were created using R (R Development Core Team 2011) igraph library (Csardi and Nepusz 2006). Each group of SNs had the following properties:

a. Group I: Same $CV(k), C(\tau_v)$ and degree distribution of the Arabidopsis M-GCN.
b. Group II: Lower $CV(k)$ than Arabidopsis M-GCN, same $C(\tau_v)$ and degree distribution.
c. Group III: Higher $CV(k)$ than Arabidopsis M-GCN, same $C(\tau_v)$ and degree distribution.

Note that the number of nodes (*n*) and number of edges (n_E) of SNs were not fixed.

iii) A similarity matrix (S_{nxn}) was created for every SN. First, a total of n^2 similarities ($s_{i,j}$) following a normal distribution were assessed forming a similarity array. Then, the array was sorted in descending order. The first n_E higher similarities were randomly assigned to pairs of nodes connected in the SN.
Consequently, a theoretical threshold τ_t^* was found when all the connected pairs of genes had a similarity assigned. In other words, the threshold τ_t^* is the n_E-*th* element of the ordered similarity array.
Remaining similarities lower than τ_t^* were assigned randomly to non-connected pairs.

iv) Using equation 4 a calculated threshold τ^* was obtained. The suitability of our method was verified comparing graphically τ^* vs. τ_t^* in each group. The absolute difference η was calculated for each group (equation S7).

$$\eta = \sum_{i=1}^{n_S} \left| \tau_i^* - \tau_{t,i}^* \right| \quad [S7]$$

In equation S7, η is the absolute difference between the theoretical thresholds τ_t^* and the calculated thresholds τ^* across the n_S simulated networks.

2.2 Evaluation of the method for threshold similarity selection

The adapted method exhibited extraordinary precision for the similarity threshold selection (see Online resource 8: Fig. S4). For all three of the SN groups, the observed threshold τ^* was close to the theoretical threshold τ_t^*. The performance is acceptable even for those SNs with higher or lower $CV(k)$ than in the *Arabidopsis* M-GCN.

Especially for the low $CV(k)$ (group 2), the SN properties are comparable to those of the M-GCNs from rice, soybean, tomato and cassava. Those $CV(k)$ for the M-GCNs from rice, soybean, tomato and cassava were relatively close to the $CV(k)$ for the SNs from group 2 (see Online resource 3: Table S2). Because the SNs in group 2 had the lowest $CV(k)$ and the lowest associated absolute

error ($\eta = 1.43$), we inferred that our methodology performs better for scale-free networks, such as those of rice, soybean, tomato and cassava.

Although the absolute error increased with a higher $CV(k)$ (group 1 and 3), the performance of this method was not affected. For that reason, the methodology proposed here could be used for networks with different topologies, not only those that are similar to the *Arabidopsis* M-GCN.

Considering these results, the absolute value of the differences between $C(\tau_v)$ and $C_r(\tau_v)$ is also suitable for the threshold selection. We applied this method (equation 4) for the entire threshold selections performed in this work.

3. Characterization of GCNs

The definition and equations of eight variables that were used to characterize GCNs are stated as follows:

i. Clustering coefficient

The clustering coefficient is the same referenced in methods section. It was calculated by equation 1.

ii. Network density

The network density is defined as the mean off-diagonal adjacency. It is a measure of cohesiveness (Horvath and Dong 2008)*:*

$$Density = \frac{\sum_i \sum_{j \neq i} a_{i,j}}{n(n-1)} \quad [S.8]$$

In equation S.7, *n* denotes the number of nodes and $a_{i,j}$ denotes the adjacency between nodes *i* and *j*.

iii. Centralization

The centralization is a measure of how central is the most central node in relation to the centrality of all nodes. Consequently, centralization is a score based on nodes centrality (Freeman 1979). Here we used the betweenness centrality measure, defined by the number of shortest paths in the graph that pass through the node, divided by the total number of shortest paths (Brandes 2001). The shortest path between two nodes is a path that interconnects both nodes with the minimum number of edges.
The betweenness centrality B_k for a node l is (Freeman 1979):

$$B_k = \sum_i \sum_j \frac{\varepsilon(i,l,j)}{\varepsilon(i,j)}, \quad i \neq l \neq k \quad [S.9]$$

In equation S.8, $\varepsilon(i,l,j)$ denotes the number of shortest paths between nodes *i* and *j* that pass through node l. $\varepsilon(i,j)$ denotes the number of shortest paths between nodes *i* and *j*.

And the GCN's centralization C_B was calculated with (Freeman 1979):

$$C_B = \frac{\sum_k^n (B_{max} - B_k)}{n^3 - 3n + 2} \quad [S.10]$$

In equation S.9, B_{max} denotes the maximum centrality of a node in the GCN. Here C_B is normalized by the network's size.

iv. Heterogeneity

The network heterogeneity is also known as coefficient of variation of node degree. It measures the variance of connectivity across the nodes (Horvath and Dong 2008):

$$Heterogeneity = \frac{\sqrt{\left[{}^1\!/_n \sum_{i=1}^n {k_i}^2\right] - \left[{}^1\!/_n \sum_{i=1}^n k_i\right]^2}}{{}^1\!/_n \sum_{i=1}^n k_i} \quad [S.11]$$

In equation S.10, k_i denotes the number of neighbors of gen *i* or node degree.

v - vi. Assortativity coefficients using different genomic data

The assortativity coefficient is a measure of how much the nodes link to other with similar characteristics (Newman 2002). Nodes are characterized based on either categorical or continuous variables. Thus, nodes share categories or values from the variables. The assortativity coefficient for categorical variables is defined by (Newman 2002):

$$r = \frac{\sum_c f_{cc} - \sum_c q_c t_c}{1 - \sum_c q_c t_c} \quad [S.12]$$

In equation S.11, f_{cd} denotes the fraction of edges connecting nodes with categories *c* and *d;* $q_c = \sum_d f_{cd}$ and $t_d = \sum_c f_{cd}$. The assortativity coefficient assumes values from -1 (dissortative network) to 1 (assortative network).

Here we calculated two assortativity coefficients using the following categorical variables, one per assortativity coefficient:

- Gene ontology (GO) annotations: They were downloaded from Phytozome (http://www.phytozome.net) and Gramene (http://www.gramene.org) using the Biomart database system. Genes were characterized by one or various GO IDs.

- PFAM annotations: They were obtained from the same public databases as GO annotations. Genes were characterized by one or various PFAM ids.

vii. *Tolerance to attacks*

We followed a common methodology to evaluate the robustness of GCNs to perturbations, here referred as tolerance to attacks, from a topological perspective (Albert and Barabasi 2001). We started from the original GCN and those nodes with the highest degree were removed sequentially, simulating an attack. To evaluate the tolerance, the average path length (l_{av}) was measured as a function of the fraction of nodes removed (*f*) from the GCN. The tolerance to attacks was defined as the critical fraction (f_c) at which l_{av} is maximum, just before that GCN breaks into isolated clusters (Albert and Barabasi 2001).

$$l_{av} = \frac{1}{n(n-1)} \sum_{i,j} a_{i,j} \quad [S.13]$$

viii. *Correlation between node degree and presence of immunity domains*

We attempted to find the dependence between node degree and presence of typical domains found in the immunity proteins. Initially, a reference dataset of genes encoding proteins involved in defense was assembled for each plant. The canonical immune protein domains (WRKY, TIR, NBS, LRR, kinase and LysM) were downloaded from PFAM (http://pfam.sanger.ac.uk/). HMMsearch was used to find these domains in the proteome of each plant (Finn et al. 2011). Those genes encoding proteins with unique domains TIR, NBS or LysM, and also genes encoding multiple different immune domains were included in the dataset. For Arabidopsis, this dataset was complemented with immunity related genes from a protein-protein interaction network (Mukhtar et al. 2011).
Afterwards, the presence or absence of immunity domains was obtained through the binary variable *b*:

$$b_i = \begin{cases} 1\ if\ gene\ i\ is\ included\ in\ the\ reference\ dataset \\ 0\ if\ gene\ i\ is\ not\ included\ in\ the\ reference\ dataset \end{cases} \quad [S.14]$$

The correlation between degree of nodes and *b* was calculated with the Kendall's tau non-parametric correlation (Kendall and Gobbons 1990).

4. Supplementary analysis of S-GCNs in the PCs space

The correlation circles of both planes show the correlations among variables (Fig. 4c and d). Heterogeneity has a negative correlation with other topological variables, which could be explained by the fact that high heterogeneity is a property of less dense, non-clustered and non-centralized GCNs that have a topology comparable to that of random networks.

The positions of S-GCNs in the planes (PC1-PC2, PC1-PC3) can be explained by the contribution of each variable to the PCs. In quadrant II of the PC1-PC2 plane (Fig. 4a), we found S-GCNs that were

influenced by high clustering coefficients, centralization and density. In quadrant III, the separation of S-GCNs was not strong, but there were networks with high assortativity coefficients from GO and PFAM.

The networks that were projected in quadrants I and VI have opposite properties from those networks in quadrants II and III (Fig. 4a). In quadrant IV, we found less clustered, dense and centralized networks that also had high heterogeneity. Quadrant I grouped a small number of differentiated networks that have negative assortativity coefficients and high values of heterogeneity.

In relation to the PC1-PC3 plane (Fig. 4b), the S-GCNs with high tolerance to attacks and immunity degree dependence were placed in quadrants I and II. The networks in quadrants III and IV have opposing properties. From this analysis, we conclude that the S-GCN differentiation by assortativity coefficients is not as good as the differentiation by other variables.

References for Supplemental Material

Albert R, Barabasi AL (2001) Statistical mechanics of complex networks. Rev Mod Phys 74:78.

Brandes U (2001) A Faster Algorithm for Betweenness Centrality. J Math Sociol 25:163–177. doi: 10.1080/0022250X.2001.9990249

Csardi G, Nepusz T (2006) The igraph software package for complex network research. InterJournal 1:1695.

Daub CO, Steuer R, Selbig J, Kloska S (2004) Estimating mutual information using B-spline functions--an improved similarity measure for analysing gene expression data. BMC Bioinformatics 5:118.

Dionisio A, Menezes R, Mendes D (2004) Mutual information: a measure of dependency for nonlinear time series. Physica A 344:326–329. doi: 10.1016/j.physa.2004.06.144

Elo LL, Järvenpää H, Oresic M, et al. (2007) Systematic construction of gene coexpression networks with applications to human T helper cell differentiation process. Bioinformatics 23:2096–2103.

Finn RD, Clements J, Eddy SR (2011) HMMER web server: interactive sequence similarity searching. Nucleic Acids Res 39:W29–W37.

Freeman L (1979) Centrality in social networks conceptual clarification. Soc Networks 1:215–239. doi: 10.1016/0378-8733(78)90021-7

Hausser J (2006) Improving Entropy Estimation and the Inference of Genetic Regulatory Networks. 33.

Horvath S, Dong J (2008) Geometric Interpretation of Gene Coexpression Network Analysis. PLoS Comput Biol 4:27.

Kendall MG, Gobbons JD (1990) Rank Correlation Methods, 5th ed. Sci Forum 3:272.

Mahanta P, Ahmed HA, Bhattacharyya DK, Kalita JK (2012) An effective method for network module extraction from microarray data. BMC Bioinformatics 13:S4. doi: 10.1186/1471-2105-13-S13-S4

Meyer PE, Lafitte F, Bontempi G (2008) minet: A R/Bioconductor Package for Inferring Large Transcriptional Networks Using Mutual Information. BMC Bioinformatics 9:461.

Mukhtar MS, Carvunis A-R, Dreze M, et al. (2011) Independently evolved virulence effectors converge onto hubs in a plant immune system network. Science (80-) 333:596–601. doi: 10.1126/science.1203659

Newman MEJ (2002) Assortative mixing in networks. Phys Rev Lett 89:5.

R Development Core Team R (2011) R: A Language and Environment for Statistical Computing. R Found Stat Comput 1:409. doi: 10.1007/978-3-540-74686-7

Scott D, Sain S (2005) Multidimensional Density Estimation. In: Rao CR, Wegman EJ, Solka JL (eds) Handb. Stat. Data Min. Data Vis. Elsevier Science, Amsterdam, pp 229–261

Soranzo N, Bianconi G, Altafini C (2007) Comparing association network algorithms for reverse engineering of large-scale gene regulatory networks: synthetic versus real data. Bioinformatics 23:1640–1647.

Zhang B, Horvath S (2005) A general framework for weighted gene co-expression network analysis. Stat Appl Genet Mol Biol 4:Article17.
