# Supplementary material for: Construction and comparison of gene co-expression networks shows complex plant immune responses
Source: PeerJ. 2014 Oct 9;2:e610. doi: 10.7717/peerj.610 (PMC4194462; doi:10.7717/peerj.610)
Supplement: Table S1 — The expression matrices dimensions, similarity thresholds, characterization variables and clusters where networks were grouped. [file peerj-02-610-s002.pdf]

Table S1. The main results for S-GCNs construction: The expression matrices dimensions, similarity thresholds, characterization variables and clusters where S-GCNs were grouped*.

id.	Plant	Sam	Gen	τ^*	Nod	Edg	CC	Cen	Het	Den	AsG	AsP	Tol	KI	Clu
1	Arabidopsis	4	4,224	0.83	112	908	0.707	0.586	0.810	7.304E-02	-8.106E-03	-1.330E-02	0.07	0.124	2
2	Arabidopsis	12	4,224	0.43	852	10,656	0.637	0.229	1.522	1.470E-02	2.532E-03	-3.354E-03	0.35	0.083	6
3	Arabidopsis	56	4,224	0.57	1,439	513,484	0.998	0.554	0.591	2.481E-01	-8.769E-04	-9.387E-04	0.00	0.022	4
4	Arabidopsis	5	4,224	0.46	378	731	0.880	0.198	1.656	5.130E-03	-9.874E-03	-3.622E-03	0.01	-0.065	7
5	Arabidopsis	16	4,224	0.29	1,400	161,715	0.985	0.806	0.687	8.257E-02	8.321E-04	-4.682E-04	0.01	-0.028	8
6	Arabidopsis	6	4,224	0.30	1,554	188,162	0.957	0.735	0.946	7.797E-02	-1.563E-03	-1.218E-03	0.03	0.040	8
7	Arabidopsis	6	4,224	0.28	522	35,473	0.898	0.366	0.614	1.304E-01	-1.954E-03	-2.596E-03	0.20	-0.059	9
8	Arabidopsis	6	4,224	0.30	1,688	244,171	0.963	0.741	0.880	8.574E-02	-6.399E-05	-7.590E-04	0.05	0.028	8
9	Arabidopsis	6	4,224	0.52	338	6,969	0.998	0.198	0.745	6.118E-02	-5.344E-03	-4.107E-03	0.87	0.044	1
10	Arabidopsis	6	4,224	0.40	920	9,945	0.649	0.271	1.597	1.176E-02	1.705E-03	-2.205E-03	0.23	0.044	6
11	Arabidopsis	27	4,224	0.86	324	2,811	0.858	0.334	1.154	2.686E-02	-5.751E-03	-2.855E-03	0.07	-0.016	7
12	Arabidopsis	8	4,224	0.69	535	4,852	0.915	0.214	1.291	1.698E-02	1.453E-03	-4.289E-03	0.17	-0.055	7
13	Arabidopsis	12	4,224	0.62	1,508	51,101	0.830	0.277	1.275	2.249E-02	1.237E-02	-5.382E-04	0.38	0.020	6
14	Arabidopsis	24	4,224	0.90	215	1,911	0.799	0.571	1.172	4.153E-02	-9.556E-03	-1.142E-02	0.23	-0.020	2
15	Arabidopsis	32	4,224	0.69	437	13,954	0.987	0.226	0.747	7.324E-02	-5.009E-03	-3.227E-03	0.29	-0.038	7
16	Arabidopsis	6	4,224	0.89	352	1,310	0.901	0.156	1.158	1.060E-02	1.638E-02	-3.495E-03	0.05	-0.008	5
17	Arabidopsis	12	4,224	0.45	1,043	18,383	0.953	0.376	1.548	1.691E-02	3.423E-03	-3.135E-03	0.17	0.091	6
18	Arabidopsis	16	4,224	0.28	708	95,194	0.990	0.589	0.368	1.902E-01	5.429E-05	-1.361E-03	0.00	-0.002	9
19	Arabidopsis	6	4,224	0.43	684	8,998	0.718	0.241	1.613	1.926E-02	-5.460E-03	-5.490E-03	0.32	-0.015	7
20	Arabidopsis	17	4,224	0.58	274	1,175	0.983	0.124	1.479	1.571E-02	-4.636E-03	-1.059E-02	0.23	0.017	7
21	Arabidopsis	16	4,224	0.45	673	4,088	0.834	0.201	1.756	9.039E-03	1.073E-02	-5.219E-03	0.13	-0.020	5
22	Arabidopsis	17	4,224	0.45	785	8,566	0.797	0.184	1.640	1.392E-02	-3.139E-03	-4.782E-03	0.26	0.046	6
23	Arabidopsis	17	4,224	0.47	784	3,067	0.917	0.079	1.538	4.996E-03	2.030E-03	-3.555E-03	0.13	0.007	7
24	Arabidopsis	17	4,224	0.48	749	13,831	0.954	0.270	1.426	2.469E-02	-1.971E-03	-3.716E-03	0.13	0.022	7
25	Arabidopsis	17	4,224	0.64	276	1,212	0.846	0.145	1.360	1.597E-02	-2.239E-03	-8.128E-03	0.03	0.041	7
26	Arabidopsis	15	4,224	0.62	668	5,191	0.723	0.157	1.371	1.165E-02	1.359E-02	-3.745E-03	0.24	-0.031	5
27	Arabidopsis	21	4,224	0.50	853	35,655	0.999	0.190	0.526	4.906E-02	1.905E-03	-1.170E-03	0.00	0.031	9
28	Arabidopsis	6	4,224	0.69	507	14,164	0.979	0.304	1.246	5.521E-02	-5.848E-03	-4.928E-03	0.26	-0.028	7
29	Arabidopsis	9	4,224	0.66	395	3,312	0.963	0.162	1.193	2.128E-02	-3.748E-03	-4.378E-03	0.17	0.008	7
30	Arabidopsis	12	4,224	0.83	104	555	0.903	0.868	1.022	5.181E-02	-1.293E-02	-1.905E-02	0.03	0.085	2
31	Arabidopsis	8	4,224	0.67	512	6,190	0.979	0.208	1.062	2.366E-02	-5.006E-03	-4.487E-03	0.38	0.005	7
32	Arabidopsis	12	4,224	0.27	662	58,645	0.884	0.421	0.680	1.340E-01	-1.614E-03	-2.090E-03	0.35	0.038	9
33	Arabidopsis	6	4,224	0.86	389	4,768	0.887	0.427	1.052	3.159E-02	8.965E-03	-4.397E-03	0.13	0.017	5
34	Arabidopsis	10	4,224	0.86	180	1,710	0.971	0.737	1.205	5.307E-02	-3.986E-03	-1.246E-02	0.01	0.006	2
35	Arabidopsis	8	4,224	0.58	670	3,807	0.799	0.689	2.190	8.493E-03	-9.411E-03	-8.692E-03	0.01	0.047	2
36	Arabidopsis	12	4,224	0.67	560	53,182	0.999	0.833	0.146	1.699E-01	-2.763E-03	-1.817E-03	0.00	0.009	9
37	Arabidopsis	3	4,224	0.67	738	9,251	0.791	0.855	1.814	1.701E-02	-3.506E-03	-4.176E-03	0.29	-0.034	7
38	Arabidopsis	24	4,224	0.53	573	7,517	0.994	0.428	1.346	2.293E-02	1.846E-03	-5.474E-03	0.17	-0.021	7
39	Arabidopsis	10	4,224	0.52	551	969	0.728	0.250	2.888	3.197E-03	-2.384E-02	-1.700E-02	0.00	-0.086	10
40	Arabidopsis	20	4,224	0.48	1,505	375,424	0.997	0.546	0.748	1.659E-01	-8.301E-04	-1.018E-03	0.55	0.011	4
41	Rice	24	6,495	0.67	782	24,078	0.934	0.307	1.204	3.942E-02	-2.849E-03	-2.899E-03	0.35	-0.020	7
42	Rice	12	6,495	0.33	885	96,638	0.905	0.634	0.672	1.235E-01	-1.334E-03	-1.618E-03	0.79	0.030	1
43	Rice	9	6,495	0.68	1,056	56,671	0.860	0.540	1.057	5.087E-02	-1.125E-03	-1.794E-03	0.47	-0.039	1
44	Rice	45	6,495	0.68	1,582	227,671	0.930	0.439	0.911	9.103E-02	-1.067E-03	-1.104E-03	0.59	0.032	1
45	Rice	8	6,495	0.49	1,221	68,353	0.998	0.202	0.474	4.589E-02	-9.863E-04	-4.867E-04	0.00	-0.019	9
46	Rice	6	6,495	0.89	214	1,005	0.779	0.134	0.791	2.205E-02	-1.164E-02	-9.645E-03	0.17	0.026	7
47	Rice	18	6,495	0.82	274	3,863	0.978	0.760	1.167	5.164E-02	-1.088E-02	-9.112E-03	0.01	-0.010	2
48	Rice	8	6,495	0.69	538	14,234	1.000	0.154	0.656	4.927E-02	-3.406E-03	-1.790E-03	0.00	0.046	9
49	Soybean	6	2,771	0.52	966	10,196	0.542	0.242	1.399	1.094E-02	7.903E-04	-9.901E-05	0.33	-0.027	3
50	Soybean	36	2,771	0.64	773	6,909	0.510	0.272	1.478	1.158E-02	-2.151E-03	-1.274E-03	0.38	0.027	3
51	Soybean	36	2,771	0.68	142	924	0.727	0.356	0.946	4.615E-02	-1.492E-02	-1.098E-02	0.36	0.112	2
52	Soybean	24	2,771	0.67	473	2,992	0.573	0.221	1.315	1.340E-02	-3.717E-03	-3.139E-03	0.23	0.032	3
53	Soybean	27	2,771	0.42	440	3,883	0.660	0.305	1.406	2.010E-02	-3.123E-03	-5.712E-04	0.30	-0.012	3
54	Tomato	160	1,481	0.61	684	53,759	0.982	0.535	0.761	1.151E-01	-2.334E-03	-2.277E-03	0.19	-0.016	9
55	Tomato	8	1,481	0.65	894	98,969	0.847	0.670	0.731	1.240E-01	-1.778E-03	-1.705E-03	0.75	-0.014	1
56	Tomato	18	1,481	0.43	432	21,099	0.998	0.481	0.888	1.133E-01	-4.068E-03	-4.180E-03	0.00	0.033	9
57	Cassava	7	747	0.93	363	1,649	0.481	0.118	1.036	1.255E-02	-5.976E-03	-6.321E-03	0.30	0.005	3
58	Cassava	33	747	0.7	456	459	0.341	0.031	0.844	2.212E-03	-1.078E-02	-5.193E-03	0.00	0.024	3
59	Cassava	54	747	0.42	261	403	0.007	0.316	1.975	5.939E-03	-1.720E-02	-1.537E-02	0.07	0.007	10

* The S-GCNs are identified with the same dataset ids from table 1. The expression matrix dimensions are the number of samples (Sam) and number of genes (Gen). The similarity threshold selected is τ^*. The variables in characterization matrix are: The number of nodes (Nod), the number of edges (Edg), the clustering coefficient (CC), the centralization (Cen), the heterogeneity or $CV(k)$ (Het) , the density (Den), the assortativity coefficient from GO (AsG), the assortativity coefficient from PFAM (AsP), the tolerance to attacks (Tol) and the correlation between node degree and presence of immunity domains (KI). The last column (Clus) identifies the clusters where S-GCNs were classified.
