# Supplementary material for: Construction and comparison of gene co-expression networks shows complex plant immune responses
Source: PeerJ. 2014 Oct 9;2:e610. doi: 10.7717/peerj.610 (PMC4194462; doi:10.7717/peerj.610)
Supplement: Table S2 [file peerj-02-610-s003.pdf]

Table S2. The characterization matrix for M-GCNs*.

Plant	Nod	Edg	CC	Cen	Het	Den	AsG	AsP	Tol	KI
Arabidopsis	1,563	4,489	0.023	0.199	2.86	1.839E-03	-1.146E-03	-8.658E-03	0.07	2.994E-02
Rice	744	3,065	0.622	0.204	1.67	5.545E-03	2.798E-03	6.581E-03	0.11	1.773E-03
Soybean	762	6,356	0.622	0.239	1.61	1.096E-02	1.797E-03	5.169E-03	0.29	2.539E-03
Tomato	674	5,794	0.816	0.169	1.27	1.277E-02	-3.813E-03	-3.808E-03	0.29	2.792E-03
Cassava	307	739	0.434	0.118	1.33	7.026E-04	-3.727E-02	-3.052E-02	0.01	1.183E-02

*The list of variables: The number of nodes (Nod), the number of edges (Edg), the clustering coefficient (CC), the centralization (Cen), the heterogeneity or $CV(k)$ (Het) , the density (Den), the assortativity coefficient from GO (AsG), the assortativity coefficient from PFAM (AsP), the tolerance to attacks (Tol) and the correlation between node degree and presence of immunity domains (KI).
