# Supplementary material for: Construction and comparison of gene co-expression networks shows complex plant immune responses
Source: PeerJ. 2014 Oct 9;2:e610. doi: 10.7717/peerj.610 (PMC4194462; doi:10.7717/peerj.610)
Supplement: Table S3 [file peerj-02-610-s004.pdf]

Table S3. The explained variance by each principal component (PC1, PC2, PC3). The correlation (PCC) between the graph variables and the network size (number of nodes in each S-GCN) is also shown.

Variable	PCC	Explained Variance (EV) (%)			Cumulated EV (%)
		PC1	PC2	PC3	
Clustering Coefficient (CC)	0.22	48.5	0.5	7.9	57
Centralization (Cen)	0.20	23.3	41.6	0.7	66
Heterogeneity (Het)	-0.12	64.5	2.1	4.1	71
Density (Den)	0.32	66.6	13.9	0.9	82
Assortativity Coefficient: GO (AsG)	0.41	21.6	46.7	1.0	69
Assortativity Coefficient: PFAM (AsP)	0.64	40.2	40.5	0.1	81
Immunity-Degree dependence (KI)	0.01	0.1	4.2	59.8	64
Tolerance to attacks (Tol)	0.18	1.9	14.4	37.9	54
