# Supplementary material for: Construction and comparison of gene co-expression networks shows complex plant immune responses
Source: PeerJ. 2014 Oct 9;2:e610. doi: 10.7717/peerj.610 (PMC4194462; doi:10.7717/peerj.610)
Supplement: Script S1 [file peerj-02-610-s009.pdf]

```
#######################
# 1. GCN Construction #
#######################

#########################
# 1.1 Expression matrix #
#########################

#Place the expression data in your working directory.
#In R, create the object "samples.names" listing your samples.
#e.g.: samples.names=c("GSM700349", "GSM700350", "GSM700351")

#Files *.CEL (Affymetrix) are preprocessed as follows:
library(affy)
#Read the data:
data=ReadAffy(filenames = samples.names)
#Normalization:
esetCEL=rma(data)
#Expression matrix construction:
E=exprs(esetCEL)

#Files *.GPR (Axon Genepix):
library(marray)
#Read the data:
rg=read.GenePix(samples.names)
#Normalization:
nrg=maNorm(rg,norm="median")
#Expression matrix construction:
E=nrg@maA

#Files *.TXT (Agilent):
library(Agi4x44PreProcess)
#Read the data:
target=made.target(samples.names)
data=read.AgilentFE(target,makePLOT=FALSE)
#Normalization:
data.n=BGandNorm(data,BGmethod="norE",NORMmethod="quantile",foreground="MeanSignal",
background="BGMedianSignal",offset=50,makePLOTpre=FALSE,makePLOTpost=FALSE)
#Deletion of non-control probes
data.p=summarize.probe(data.n,makePLOT=FALSE,target)
#Expression matrix construction:
E=data.p$R

########################
#1.2 Similarity Matrix #
########################

#The similarity matrix is saved in files .bin and .des.
#Bigmatrix files:
library(bigmemory)
n=nrow(E)
x=filebacked.big.matrix(n*(n-1)/2, 1, type="double",
backingfile="similaritypair.bin", descriptorfile="similaritypair.desc") #Por MI
S=filebacked.big.matrix(n, n, type="double", backingfile="similarity.bin",
descriptorfile="similarity.desc")

#Select the number of processors for a parallel process:
library(doParallel)
Sys.getenv('NUMBER_OF_PROCESSORS')
cl <- makeCluster(5)
registerDoParallel(cl)

#Discretize the expression matrix:
```

```
Ed=discretize(t(E), disc="globalequalwidth",nbins=floor(1+log2(dim(E)[2])))

#Load the bigmatrix:
desc<-dget("similaritypair.desc")
x=attach.big.matrix(desc);flush(x)

#Function to calculate the Mutual information ("shrink" entropy estimator):
mimodificada=function(X,Y,ensy){
  U <- cbind(Y, X)
  Hx <- .Call("entropyR",X, ensy, 1, 2, DUP = FALSE, PACKAGE = "infotheo")
  Hy <- .Call("entropyR",Y, ensy, 1, 2, DUP = FALSE, PACKAGE = "infotheo")
  Hyx <- .Call("entropyR",U, ensy, 2, 2, DUP = FALSE, PACKAGE = "infotheo")
  res <- Hx + Hy - Hyx
  res
}

#The similarities between pairs of genes i and j are calculated as follows:
n=dim(Ed)[2]#Number of genes
ens=dim(Ed)[1]#Number of samples
k=1#Starting gene
paso=100#Step
while(k<n){
  print(k)
  if((k+paso)>n){paso=1}
  lista<-foreach(i=k:(k+paso), .export=c("mutinformation")) %dopar% {
    unlist(lapply((i+1):n, function(j) {mimodificada(Ed[,i],Ed[,j],ens)}))
  }
  IM=unlist(lista)
  IM[which(IM<0)]<-0
  end=sum(seq(n-1,n-(k+paso)))
  start=sum(seq(n-1,n-k))-(n-(k+1))
  x[start:end,1]<-sqrt(1-exp(-2*IM))
  flush(x)
  remove(lista)
  gc()
  k=k+paso
}

#Load the similarity matrix:
desc<-dget("similarity.desc")
S=attach.big.matrix(desc)
dim(S);flush(S)

#Fill the similarity matrix:
start=1
for(i in 1:(n-1)){
  end=start+(n-i-1)
  S[(i:n),i]<-c(1,x[(start:end),1])
  start=end+1
  flush.console()
}
S[n,n]=1;flush(S)

###########################
#1.3 Similarity threshold #
###########################

#Create matrices to save:
#The local clustering coefficient Ci for each similarity threshold.
C=filebacked.big.matrix(n, 100, type="double", backingfile="ci.bin",
descriptorfile="ci.desc")
#The number of neighbors of gene i or node degree (ki) for each similarity
threshold.
```

```
K=filebacked.big.matrix(n, 100, type="double", backingfile="ki.bin",
descriptorfile="ki.desc")

#Similarity thresholds evaluated:
(ltaos=seq(0.99,0.01,by=-0.01))

#Calculate the node degree and the local clustering coefficient as the similarity
threshold change:
for(tao in ltaos){
  print(tao)
  ##Adjacency matrix
  A=matrix(0,nrow=n,ncol=n)
  ##Fill the adjacency matrix (equation 1):
  for(i in 1:n){
    v.unos=mwhich(S,cols=i,vals=tao,comps='gt')
    v.ceros=(1:n)[((1:n)%in%v.unos)==FALSE]
    A[v.unos,i]<-1
    A[v.ceros,i]<-0
  }
  ##Calculate the ki and Ci:
  Ag=graph.adjacency(A,mode="undirected")
  remove(A);gc()
  Cv=transitivity(Ag,type="local")#Local clustering coefficient
  Kv=degree(Ag,loops=FALSE)#Node degree
  desc<-dget("ki.desc");K=attach.big.matrix(desc)
  desc<-dget("ci.desc");C=attach.big.matrix(desc)
  K[,round(tao*100,0)]<-Kv;C[,round(tao*100,0)]<-Cv;flush(K);flush(C)
  remove(K);remove(C)
}

#Calculate the observed clustering coefficient (Cr) in the GCN the expected
clustering coefficient (Co)
desc<-dget("ki.desc");K=attach.big.matrix(desc)
desc<-dget("ci.desc");C=attach.big.matrix(desc)
Cr=Co=rep(0,100);(ltaos=seq(0.01,0.99,by=0.01))
for(i in round(ltaos*100,0)){
  gn<-which(K[,i]>=1)
  kn=length(gn)#Number of genes in the network
  k1=1/kn*sum(K[gn,i])#Variable in equation 3
  k2=1/kn*sum(K[gn,i]^2)#Variable in equation 3
  Co[i]=((k2-k1)^2)/(kn*k1^3) #The expected clustering coefficient for a randomized
GCN
  if(kn==0){Co[i]=0}
  gn<-which(K[,i]>1)
  kn=length(gn)#Number of genes with ki>1
  Cr[i]=1/kn*sum(C[gn,i])#The observed clustering coefficient (equation 2)
  if(kn==0){Cr[i]=0}
}

#Find the first local maximum of the curve:
(ltaos=seq(0.01,0.99,by=0.01))
dif=runmed(abs(Cr-Co),k=23,endrule="constant")[1:100]
plot(ltaos,abs(Cr-Co)[ltaos*100],t="l",xlab="Threshold",ylab="|C-Co|")

#Identify the maximum and click on it:
tao=identify(ltaos,abs(Cr-Co)[ltaos*100],n=1)/100

#######################
#1.4 Adjacency matrix #
#######################

#Create a bigmatrix file for the adjacency matrix:
A=filebacked.big.matrix(n, n, type="double", backingfile="ady.bin",
```

```
descriptorfile="ady.desc")

#Fill the adjancency matrix:
for(k in 1:n){
  v.unos=mwhich(S,cols=k,vals=tao,comps='gt')
  v.ceros=(1:n)[((1:n)%in%v.unos)==FALSE]
  A[v.unos,k]<-1
  A[v.ceros,k]<-0
  print(k)
}
flush(A)

#######################
#2. GCNs Comparison   #
#######################

#######################
#2.1 Characterization #
#######################

library(doParallel);library(Kendall);library(bigmemory);library(igraph);library(robf
ilter);library(minet);library(ade4)

#Load the bigmatrix files from section 1.3 and 1.4
desc<-dget("ki.desc");K=attach.big.matrix(desc)
desc<-dget("ci.desc");C=attach.big.matrix(desc)
desc<-dget("ady.desc",sep="")
A=attach.big.matrix(desc)
A=as.matrix(A)

#Delete non-connected nodes from adjacency matrix:
A=A[which(K[,round(tao*100,0)]>0),which(K[,round(tao*100,0)]>0)]
n=dim(A)[1]
Ag=graph.adjacency(A,mode="undirected")

#Create an array to save the graph variables:
Zr=rep(NA,10)

#--Number of nodes:
Zr[1]=length(which(K[,round(tao*100,0)]>0))

#--Number of edges:
Zr[2]=sum(K[,round(tao*100,0)])/2

#--Clustering coefficient:
gn<-which(K[,round(tao*100,0)]>1)
kn=length(gn)
Cr=1/kn*sum(C[gn,round(tao*100,0)])
if(kn==0){Cr=0}
Zr[3]<-Cr

#--Heterogeneity:
gn<-which(K[,round(tao*100,0)]>=1)
kn=length(gn)
k1=1/kn*sum(K[gn,round(tao*100,0)])
k2=1/kn*sum(K[gn,round(tao*100,0)]^2)
Zr[5]=sqrt(k2-k1^2)/k1

#--Density:
Zr[6]=sum(A[upper.tri(A)])/(n*(n-1))

#--Centralization:
kmax=max(K[,round(tao*100,0)])
```

```
Zr[4]=(n/(n-2))*(kmax/(n-1)-Zr[6])

#--Assortativity:

##You must load a file of functional annotations (ids.) with the following structure
(3 columns):
#<gene> <go>    <pfam>
#AT5G47220      GO5634  PF00847
#AT1G71920      GO9507  PF00155
#AT5G23960      g0          PF01397
#AT3G45248      GO12505 0

#Use g0 and 0 for genes without GO and PFAM annotations, respectively.

##List the genes in your adjacency matrix:
namegen=rownames(E)
namegen=namegen[which(K[,round(tao*100,0)]>0)]

##The assortativity coefficient from the GO:
go=asortatividad$go[match(namegen,asortatividad$gene)]
go[is.na(go)]<-"g0"
maxcat=length(names(table(go)))
for (i in 2:maxcat){
  go[which(go==names(table(go))[i])]<-(i-1)
}
if(names(table(go))[maxcat]=="g0"){
  nos=sum(go=="g0")
  go[go=="g0"]<-seq(maxcat,(maxcat+nos-1))
} else {go[go==names(table(go))[maxcat]]<-maxcat}
go=as.integer(go)

Zr[7]<-assortativity.nominal(Ag,go)

##The assortativity coefficient from the PFAM:
pfam=asortatividad$pfam[match(namegen,asortatividad$gene)]
pfam[is.na(pfam)]<-"0"
maxcat=length(names(table(pfam)))
for (i in 2:maxcat){
  pfam[which(pfam==names(table(pfam))[i])]<-(i-1)
}
if(names(table(pfam))[1]=="0"){
  nos=sum(pfam=="0")
  pfam[pfam=="0"]<-seq(maxcat,(maxcat+nos-1))
} else {pfam[pfam==names(table(pfam))[maxcat]]<-maxcat}
pfam=as.integer(pfam)

Zr[8]<-assortativity.nominal(Ag,pfam)

#--The correlation between the node degree and the presence of immunity domains

##You must load a list of genes encoding proteins involved in immunity.
##Name the object "esen". e.g. esen=c("AT1G01560", "AT1G06460", "AT1G08720")

es=rep(0,length(namegen))
es[namegen%in%esen]<-1
Zr[9]=Kendall(K[which(K[,round(tao*100,0)]>=1),round(tao*100,0)],es)$tau[1]

#--The tolerance to attacks
n=dim(A)[1]
percentages=c(seq(0.01,0.99,0.01))#Fraction of nodes removed
avo=average.path.length(Ag,directed=FALSE, unconnected=TRUE)#The average path length
orderfilter=n-rank(degree(Ag),ties.method="first") #Nodes with the highest degree
that will be removed sequentially
```

```
avpath<-foreach(i=percentages, .export=c("graph.adjacency","average.path.length"))
%dopar% {
  filter=which(orderfilter<round(i*n,0))
  Ai=graph.adjacency(Ag[-filter,-filter],mode="directed")
  average.path.length(Ai,directed=FALSE, unconnected=TRUE)#The average path length
as a function of the fraction of nodes removed
}
avpath=c(avo,unlist(avpath))
avpath[is.na(avpath)]<-1
Zr[10]=c(0,percentages)[which(avpath==max(avpath))][1]#The critical fraction at
which the average path length is maximum


#######################
#2.2 ACP              #
#######################

#Create a characterization matrix for all your GCNs. Name it "Z".
#Performs a principal component analysis:
library(adegenet);library(ade4)
pca=dudi.pca(as.data.frame(Z),scannf=TRUE)

#Plot correlation circles:
s.corcircle(pca$co)
add.scatter.eig(pca$eig, 3, 1, 2, posi="bottomright")
#Plot principal components:
xax=1;yax=2#PC1-PC2
plot(pca$li[,xax],pca$li[,yax],pch=19,ylab=paste("PC",yax),xlab=paste("PC",xax))
abline(v=0,lty=2,col="gray");abline(h=0,lty=2,col="gray")
pointLabel(pca$li[,c(xax,yax)],label=as.character(seq(1,nrow(Z))),method="GA",
doPlot=TRUE,cex=0.5,col="black",font=2)

#Find clusters:
find.clusters(Z,choose.n.clust=TRUE,criterior="max",stat="BIC",n.pca=10,
max.n.clust=50)
```
